# Clinical significance of CD161^+^CD4^+^ T cells in the development of chronic antibody-mediated rejection in kidney transplant recipients

**DOI:** 10.1371/journal.pone.0200631

**Published:** 2018-07-16

**Authors:** Kyoung Woon Kim, Bo-Mi Kim, Kyoung Chan Doh, Chan-Duck Kim, Kyung Hwan Jeong, Sang-Ho Lee, Chul Woo Yang, Byung Ha Chung

**Affiliations:** 1 Convergent Research Consortium for Immunologic Disease, Seoul St. Mary's Hospital, Seoul, Korea; 2 Department of Internal Medicine, College of Medicine, The Catholic University of Korea Seoul, Korea; 3 Department of Internal Medicine, Kyungpook National University School of Medicine, Daegu, Korea; 4 Department of Internal Medicine, College of Medicine, Kyung Hee University, Seoul, Korea; Seoul National University College of Pharmacy, REPUBLIC OF KOREA

## Abstract

In this study, we investigated whether CD161^+^CD4^+^ T cells can reflect the Th17 pathway in kidney transplant recipients (KTRs) and investigated the clinical significance of this cell type in chronic antibody-mediated rejection (cAMR) in KT. First, we investigated the relationship between CD161^+^CD4^+^ T and Th17 cells by flow cytometry and microarray analysis in an *in vitro* study. Second, we compared the proportion of T cell subsets including CD161^+^CD4^+^ T cells in cAMR (n = 18), long-term graft survival (LTGS) (n = 46), and interstitial fibrosis/tubular atrophy (IF/TA) (n = 22). We compared CD161^+^ cell infiltration between cAMR and IF/TA and also examined the effect of CD161^+^ T cells on human renal proximal tubular epithelial cells (HRPTEpiC). In flow cytometry, the proportion of CD161^+^CD4^+^ T cells showed a significant correlation with the proportion of Th17 cells. In microarray analysis, transcripts associated with the Th17 pathway such as *IL18RAP*, *IL-18R1*, *IL23R*, *IL12RB2*, *RORC*, *TBX21*, and *EOMES* were upregulated in CD161^+^ cells compared with CD161^-^ cells. In an *ex vivo* study, only CD161^+^CD4^+^ T cells showed a significant increase in the cAMR group compared with IF/TA and LTGS groups. In allograft tissue, CD161^+^ cells showed a higher level of infiltration in the cAMR group than the IF/TA group. Lastly, CD161^+^ T cells increased the production of inflammatory cytokines from HRPTEpiC in a dose-dependent manner. This study suggests that monitoring of CD161^+^ T cells can be useful to detect the progression of cAMR.

## Introduction

CD4^+^ T cells that produce the pro-inflammatory cytokine IL-17 have been recognized as a T cell subset distinct from Th1 and Th2, termed Th17 cells [1, 2]. Some previous studies suggested that activation of Th17 cells may play a significant role in the development of allograft injury in organ transplantation [3–7]. In our previous study, we showed that increased Th17 infiltration in rejected allograft tissue was associated with more severe allograft rejection or adverse allograft outcome after the episode of rejection [8–10]. In addition, we found that levels of Th17 cells, especially IL-17-producing effector memory T cells, were increased in kidney transplant recipients (KTRs) with chronic allograft dysfunction compared with KTRs with stable allograft function with long-term follow-up [11].

Moreover, previous studies recognized that Th17 cell clones show specific expression of CD161, which is a C-type lectin-like receptor [12]. CD161 is a marker of human memory Th17 cells and CD4^+^CD161^+^ T cells can be differentiated into pathogenic Th17 cells, which exhibit inflammatory activity in various types of disease [13, 14]. In regard to the clinical significance, CD161^+^ T cells from the inflammatory infiltrate in psoriasis and inflammatory bowel disease were enriched for IL-17 producers. In addition, CD161^+^ T cells are also a predictive marker for acute graft-versus-host disease after hematopoietic stem cell transplantation [15]. All of these findings suggest that CD161^+^ T cells may share the characteristics of Th17 cells, and therefore this cell type may have a pathologic role in the development of immunologic disorders mediated by Th17 cells. However, in the field of kidney transplantation the significance of CD161^+^ T cells has been scarcely reported and their role has not been clearly demonstrated [16].

In this regard, the aim of this study was to investigate whether CD161^+^ T cells can reflect activation of the Th17 cell pathway and to investigate the clinical significance of CD161^+^ T cells in kidney transplantation. For this, we analyzed the relationship between CD161^+^ T cells and Th17 cells in *in vitro* and *ex vivo* studies and also investigated whether CD161^+^ T cells in the peripheral blood or allograft tissue show clinical significance in chronic antibody-mediated rejection (cAMR).

## Materials and methods

### Patients and clinical information

We examined the association between CD161^+^CD4^+^ T cells and Th17 cells in an *in vitro* study using peripheral blood from healthy subjects (n = 3) for flow cytometry and microarray analysis and in an *ex vivo* study using peripheral blood from 39 KTRs with stable allograft function.

In an *ex vivo* study to compare CD4^+^ T cell subsets among clinical groups, peripheral blood mononuclear cell (PBMC) samples were chosen from the ARTKT-1 (assessment of immunologic risk and tolerance in kidney transplantation) study, a cross-sectional sample collection study of KTRs who received kidney allograft biopsy or who had long-term allograft survival (LTGS) with stable allograft function (MDRD eGFR ≥ 50 mL/min/1.73 m^2^) over 10 years at five different transplant centers (Kyoung Hee University Hospital at Gangdong, Kyung Hee University Hospital, Kyungpook National University Hospital, Seoul St. Mary's Hospital of Catholic University of Korea) from August 2013 to July 2015. Among PBMC samples collected for the ARTKT-1 study, we selected a total of 86 samples from 18 patients with cAMR and 22 patients with interstitial fibrosis and tubular atrophy (IF/TA) on allograft biopsy with Banff classification assessed by a single pathologist [17] and 46 patients with LTGS for the present study.

All participants provided written informed consent in accordance with the Declaration of Helsinki. This study was approved by the local Institutional Review Board of Seoul St. Mary’s Hospital (KC13TNMI0701) and registered in Clinical Research Information Service (KCT0001010).

### Isolation and flow cytometric analysis of peripheral blood mononuclear cells

Peripheral blood was collected for analysis of immune cell profiles and processed as follows. PBMCs were prepared from heparinized blood by Ficoll–Hypaque (GE Healthcare, PA) density-gradient centrifugation. Cell cultures were performed as described previously [18]. In brief, the cell suspension was adjusted to a concentration of 10^6^ cells/ mL in RPMI1640 medium supplemented with 10% fetal calf serum, 100 U/mL penicillin, 100 mg/mL streptomycin, and 2 mM l-glutamine. The cell suspension (1 ml) was dispensed into 24-well multiwell plates (Nunc, Roskilde, Denmark).

### In vitro study

Isolated PBMC cells (5×10^5^) from healthy individuals were incubated under appropriate conditions for 48 hours. To induce Th0 differentiation, PBMCs (5×10^5^) were incubated for 48 hours with anti-CD3 (1 μg/mL) and anti-CD28 (1 μg/mL) antibodies (BD Biosciences, San Diego, CA, USA). To induce Th17 differentiation, PBMCs (5×10^5^) were incubated for 48 hours with anti-CD3 (1 μg/mL) and anti-CD28 (1 μg/mL) antibodies (BD Biosciences), IL-1β (20 ng/mL) (R&D Systems, Inc. Minneapolis, MN, USA), IL-6 (20 ng/mL) (R&D Systems), IL-23 (20 ng/mL) (R&D Systems), and IFN-γ–neutralizing antibody (2 μg/mL) (R&D Systems), and IL-4-neutralizing antibody (2 μg/mL) (R&D Systems).

### Flow cytometric analysis

For cytokine detection at the single-cell level, PBMCs were stimulated with 50 ng/mL phorbol myristate acetate (PMA) and 1 μg/mL ionomycin in the presence of GolgiStop (BD Biosciences) for 4 hours. For surface staining, cells were stained with combinations of mAbs to the following proteins: CD4–PE/Cy7 (RPA-T4, IgG1; BioLegend, San Diego, CA); CD161-APC (HP-EG10, IgG1; eBioscience, San Diego, CA, USA); CD45RA-FITC (HI100, IgG2b, κ; Pharmingen, San Diego, CA, USA). Staining for chemokine receptors was performed using anti-CCR7 (3D12, IgG2a, κ) mouse mAbs (Pharmingen). Cells were washed, fixed, permeabilized, and stained with mAbs to IL-17 (PE, eBio64dec17, IgG1,κ; eBioscience) to detect intracellular cytokines. Appropriate isotype controls were used for gate setting for cytokine expression. Cells were analyzed on a FACS Calibur flow cytometry system (BD Biosciences).

### Microarray analysis

#### Isolation of CD161^+^ or CD161^-^ cells and extraction of RNA

CD161^+^ T cells were purified by CD161-APC (HP-EG10, IgG1; eBioscience). The cells were sorted using a FACS Aria device (Becton Dickinson) or a MoFlo cell sorter (Beckman Coulter) to isolate CD161^+^ and CD161^-^ T cells. mRNA was extracted from CD161^+^ and CD161^-^ T cells using the ReliaPrep™ RNA Miniprep Systems (Promega Corporation, Madison, WI, USA), according to the manufacturer’s instructions.

#### RNA quality check

RNA purity and integrity were evaluated by ND-1000 Spectrophotometer (NanoDrop, Wilmington, DE, USA).

#### Affymetrix Whole Transcript Expression array method

The Affymetrix Whole Transcript Expression array process was performed according to the manufacturer's protocol (GeneChip WT Pico Reagent Kit). cDNA was synthesized using the GeneChip WT Pico Amplification kit as described by the manufacturer. The sense cDNA was fragmented and biotin-labeled with TdT (terminal deoxynucleotidyl transferase) using the GeneChip WT Terminal labeling kit. Approximately 5.5 μg of labeled DNA target was hybridized to the Affymetrix GeneChip Human 2.0 ST Array at 45°C for 16 hours. Hybridized arrays were washed and stained on a GeneChip Fluidics Station 450 and scanned on a GCS3000 Scanner (Affymetrix). Signal values were computed using the Affymetri® GeneChip™ Command Console software.

#### Immunohistochemistry (IHC) for CD161^+^ cells in allograft tissue

Eight cases of allograft biopsy from the cAMR group and IF/TA group respectively were examined for CD161^+^ cell infiltration. Paraffin sections were immersed in three changes of xylene and hydrated using a graded series of alcohols. Antigen retrieval was routinely performed by immersing the sections in sodium citrate buffer (pH 6.0) in a microwave for 15 min. The sections were depleted of endogenous peroxidase activity by addition of methanolic hydrogen peroxide and were blocked with normal serum for 30 min. After overnight incubation with polyclonal antibodies against CD161 (Abcam, Cambridge, UK), the samples were incubated with the secondary antibodies, biotinylated with anti-IgG for 20 min, and incubated with a streptavidin-peroxidase complex (Vector, Peterborough, UK) for 1 hour. After incubation with 3, 3-diaminobenzidine (Dako, Glostrup, Denmark) and counterstaining with hematoxylin, samples were photographed with an Olympus photomicroscope (Tokyo, Japan). Positivity for each IHC stain was examined in a blinded manner with respect to the clinical information.

#### Analysis of IHC results

IHC staining was analyzed by counting the total number of infiltrating cells that expressed CD161^+^ in the cortex. The area of cortex was measured with a loupe and the data were expressed as the number of cells/mm^2^. Counting of CD161^+^ cells was performed by HistoQuest Experiment (TissueQuest Software, TissueGenostics, Vienna, Austria) [9, 10].

### Co-culture of human renal proximal tubular epithelial cell (HRPTEpiC) line and isolated CD161^+^ T cells

For co-culture experiments with CD161^+^ T cells, HRPTEpiC were seeded in 24-well plates at 2×10^4^ cells/well with 1 mL of medium. Isolated CD161^+^ T cells (2×10^5^ cells/well) were added to the HRPTEpiC monolayers and the culture plates were incubated for 72 hours. On day 3, the harvested cells were examined for proliferation using a FACSCalibur flow cytometer (BD Biosciences). The culture supernatants were collected and stored at −80°C until assayed. All cultures were set up in triplicate. The levels of cytokines IL-6 and IL-8 in the culture supernatants from HRPTEpiC were measured by sandwich ELISA (R&D Systems) according to the manufacturer’s instructions. Absorbance at 405 nm was measured using an ELISA microplate reader (Molecular Devices).

### Raw data preparation and statistical analysis

Statistical analysis was performed using SPSS software (version 16.0; SPSS Inc., Chicago, IL, USA). The comparison of values among groups was performed using one-way analysis of variance. For categorical variables, chi-square frequency analysis was used. The results are presented as mean ± standard deviation (SD). *P* values < 0.05 were considered significant. For microarray analysis, raw data were extracted automatically in the Affymetrix data extraction protocol using software provided by Affymetrix GeneChip® Command Console® Software (AGCC). After importing CEL files, the data were summarized and normalized with the robust multi-average (RMA) method implemented in Affymetrix® Expression Console™ Software (EC). We exported the results with gene level RMA analysis and performed differentially expressed gene (DEG) analysis. Statistical significance of the expression data was determined using fold change and LPE test in which the null hypothesis was that no difference exists among groups. False discovery rate (FDR) was controlled by adjusting the P value using Benjamini-Hochberg algorithm. For a DEG set, hierarchical cluster analysis was performed using complete linkage and Euclidean distance as a measure of similarity. Gene-Enrichment and Functional Annotation analysis for significant probe list was performed using Gene Ontology (GO) (www.geneontology.org/) and KEGG (www.genome.jp/kegg/). All data analysis and visualization of differentially expressed genes was conducted using R 3.1.2 (www.r-project.org).

## Results

### The relation between CD161^+^CD4^+^ T cells and Th17 cells

**Fig 1:**
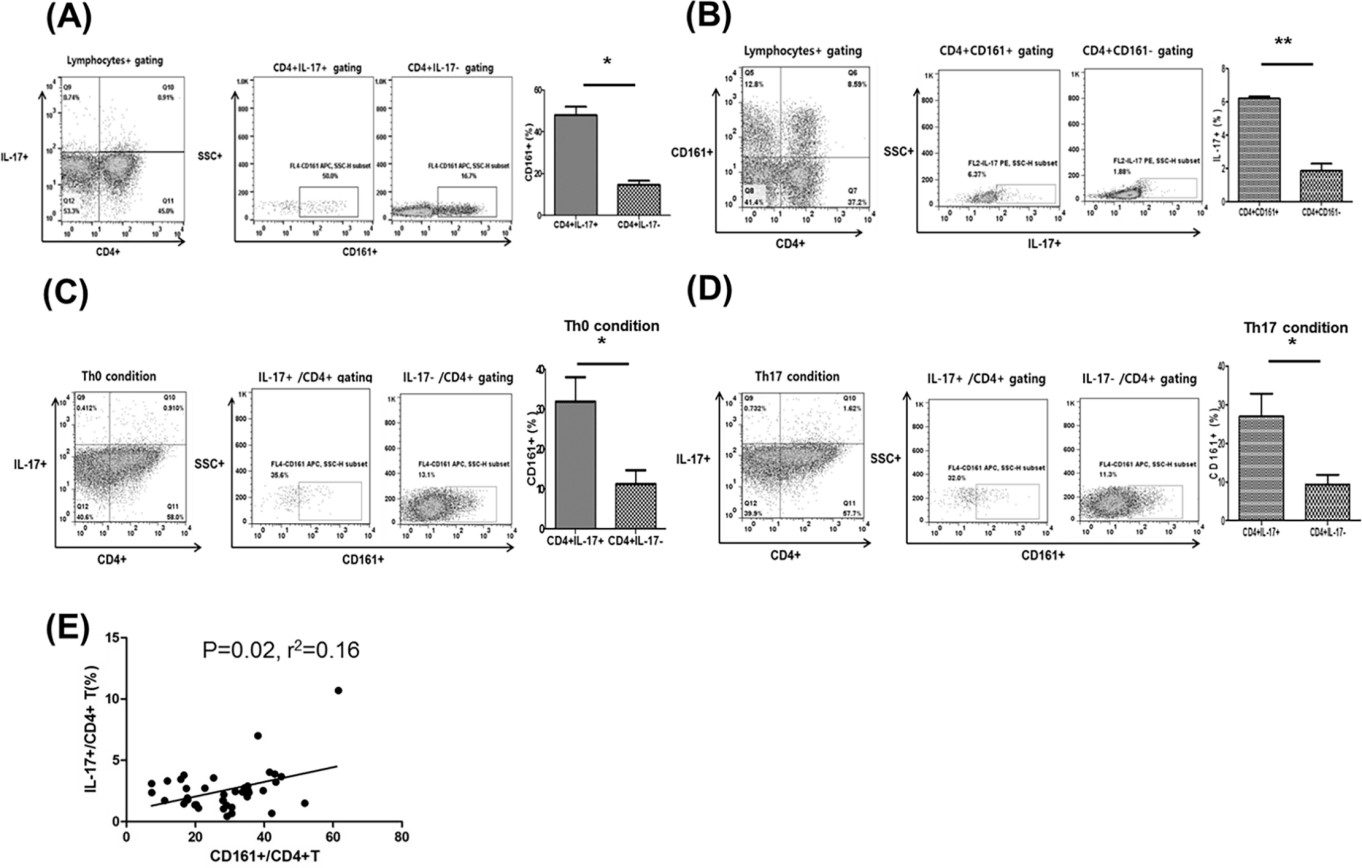
Association between CD161^+^ T cells and Th17 cells. PBMCs were stained with anti-CD4 PE-cy7, anti-CD161 APC, and anti-IL-17 PE antibodies. Lymphocytes were gated for further analysis. (A) Proportion (%) of CD161^+^/CD4^+^ IL-17^+^ T cells. (B) Proportion (%) of IL-17^+^/CD4^+^ CD161^+^ T cells in PBMCs. (C) PBMCs were cultured for 48 hours under Th0 differentiation conditions and the proportion (%) of CD161^+^/CD4^+^ IL-17^+^ T cells in PBMCs was analyzed. (D) PBMCs were cultured for 48 hours under Th17 differentiation conditions and the proportion (%) of CD161^+^/CD4^+^ IL-17^+^ T cells in PBMCs was analyzed. (E) The proportion (%) of CD161^+^/CD4^+^ T cells showed a significant positive correlation with the proportion (%) of IL-17^+^/CD4^+^ T cells (P = 0.02, r2 = 0.16). *p<0.05 for each comparison.

In the *ex vivo* study, the proportion of CD161^+^ cells was significantly higher in the IL-17^+^ fraction than the IL-17^-^ fraction within CD4^+^ gating and the proportion of IL-17^+^ cells was significantly higher in the CD161^+^ fraction than the CD161^-^ fraction (*p*<0.05 for both; Fig 1A and 1B). In an *in vitro* study with Th0 or Th17 polarization conditions, the proportion of CD161^+^ cells was significantly higher among IL-17^+^ cells than in the IL-17^-^ fraction within CD4^+^ gating and the proportion of IL-17^+^ cells was significantly higher in the CD161^+^ fraction than the CD161^-^ fraction (*p*<0.05 for both; Fig 1C and 1D). In an *ex vivo* analysis of CD161^+^CD4^+^ T cells and Th17 cells in 39 KTRs, the proportion of CD161^+^CD4^+^ T cells showed a significant correlation with the proportion of IL-17^+^CD4^+^ T cells (*p* = 0.02, r^2^ = 0.16) (Fig 1E). In the Fig 1, we found that CD161^+^CD4^+^ T cells showed a significant correlation with IL-17 producing CD4^+^ T cells (Th17 cells) in *ex vivo* and *in vitro* studies.

### Phenotypic and transcriptional profile of CD161^+^ T cells in comparison with CD161^-^T cells

**Fig 2:**
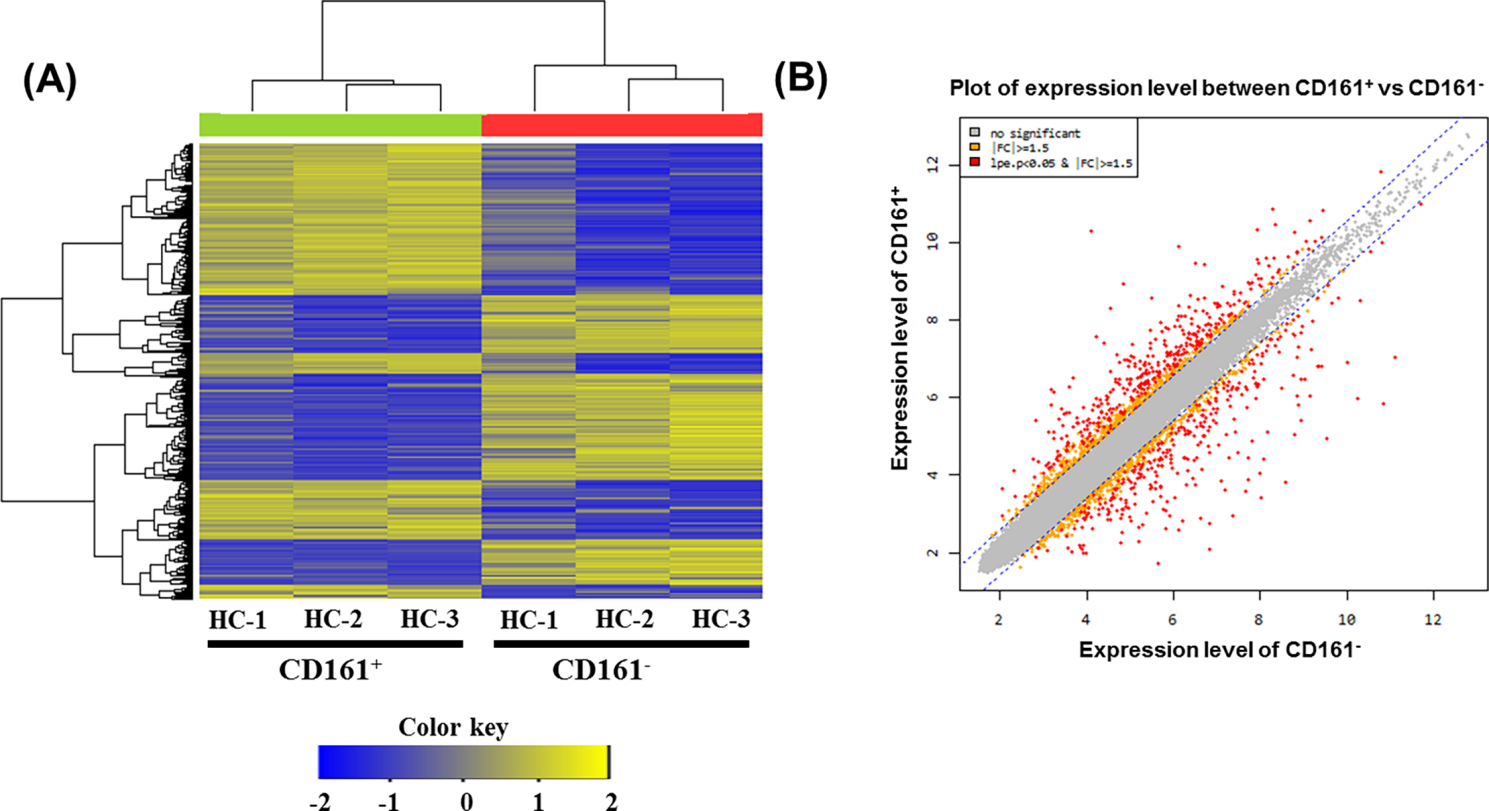
Gene expression in CD161^+^ T cells and CD161^-^ T cells using microarray. (A) Hierarchical clustering of gene expression in CD161^+^ T cells and CD161^-^ T cells. Heatmap is showing 691 significantly (*p*<0.05) differentially expressed transcripts between CD161^+^ and CD161^-^ T cells in three donors. The 691 genes were selected for this analysis by the criteria described in ‘‘Materials and Methods”. Expression levels are normalized for each gene and shown by color, with yellow representing high expression and blue representing low expression. (B) Scatter plot of expression level between CD161^+^ and CD61^-^ T cells.

We performed microarray analysis on CD161^+^ T cells compared with CD161^-^ T cells from the same donors (n = 3) (Fig 2A) and identified 574 differentially expressed genes. Moreover, comparison of the leading-edge gene set (the core set of genes that account for this enrichment) from each T cell population distinguished a core of 330 upregulated (see S1 Table) and 244 downregulated (see S2 Table) genes that were commonly enriched in all CD161-expressing T cells and therefore define the CD161-associated transcriptional signature. Genes whose expression levels were higher than the assumed threshold (upregulated >1.5-fold and downregulated <1.5-fold) were visualized using the scatter plot method, selected, and listed in the tables. Among the investigated upregulated genes, the expression of 17 genes increased (upregulated >5-fold) and expression of 38 genes decreased (downregulated <5-fold) in the CD161^+^ expressing T cells. Among the increased genes, expression of five genes increased very significantly, by >10-fold; these genes were *KLRB1*, *IL18RAP*, *SH2D1B*, *TRDJ3*, and *TRDJ4*. The expression of the following 17 genes decreased very significantly: *ZCWPW2*, *IGHD*, *IGHV1-18*, *JCHAIN*, *IGHV3-33*, *IGLC7*, *IGLC2*, *CD22*, *IGHV3-48*, *IGHA1*, *IGKV2-24*, *IGKV3D-15*, *MS4A1*, *BLNK1*, *IGHG1*, *IGHV4-34*, and *KIAA0226L*. Six genes that were upregulated in the CD161^+^ expressing T cells were associated with Th17 cells: *IL18RAP*, *IL-18R1* (= IL-18R alpha), *IL23R*, *IL12RB2*, *RORC*, *TBX21*, and *EOMES*. GO analysis tools for pathway analysis were used to investigate the biological process, cellular component, molecular function analysis of CD161^+^ T cells in comparison with CD161^-^ T cells (see S1 Fig). Microarray analysis showed that transcripts associated with the Th17 pathway were upregulated in CD161^+^ T cells.

### Comparison of the proportion of CD161^+^ T cells between chronic antibody-mediated rejection group and control groups

Table 1 shows the baseline clinical characteristics of included patient populations. There was no significant difference in the mean age of patients between LTGS and IF/TA groups, but patients in the cAMR group were younger than those in the LTGS group. Post-transplant duration was longer in the LTGS group than other two groups, but did not differ between cAMR and IF/TA groups. Allograft function assessed by MDRD eGFR was best in the LTGS group followed by the IF/TA group, and was worst in the cAMR group (*p*<0.01). Hemoglobin level was significantly higher in the LTGS group than the other two groups (*p*<0.01), and did not differ between cAMR and IF/TA groups.

**Table 1:** Baseline characteristics of the patient populations.

	cAMR (n = 18)	LGS (n = 46)	IF/TA (n = 22)	*P* for trend
**Age (year)**	50.3 ± 9.5*	56.8 ± 9.8	51.9 ± 9.4	0.03
**Male, n (%)**	10 (55.6)	18 (39.1)	14 (63.6)	0.14
**Post-transplant, months**	85.9 ± 59.0*	186.4 ± 75.9	54.8 ± 58.7	<0.01
**MDRD eGFR (mg/dL) at sampling**	26.9 ± 12.8*	72.1 ± 16.7	40.1 ± 15.2	<0.01
**Donor type (LD / DD), n (%)**	12 (67)/ 6 (33)	36 (78) / 13 (22)	12 (55) / 10 (45)	0.13
**HLA mismatch number**	3.0 ± 1.6	2.6 ± 1.3	3.1 ± 1.72	0.47
**Positive for HLA-DSA, n (%)**	14 (78)*^,^^†^	0 (0)	2 (9)	<0.01
**Hemoglobin (g/dL)**	10.9 ± 2.1*	13.0 ± 1.5	11.0 ± 1.7	<0.01
**Spot urine protein/creatinine (g/g)**	1.4 ± 3.0*^,^^†^	0.09 ± 0.2	0.09 ± 0.04	<0.01

Abbreviations; cAMR, chronic antibody mediated rejection; TCMR, t cell mediated rejection; LTGO; long term good outcome; IF/TA, interstitial fibrosis/tubular atrophy; HLA-DSA, anti-human leukocyte antigen donor specific antibody

*P<0.05 for LGS

†P<0.05 for IF/TA

**Fig 3:**
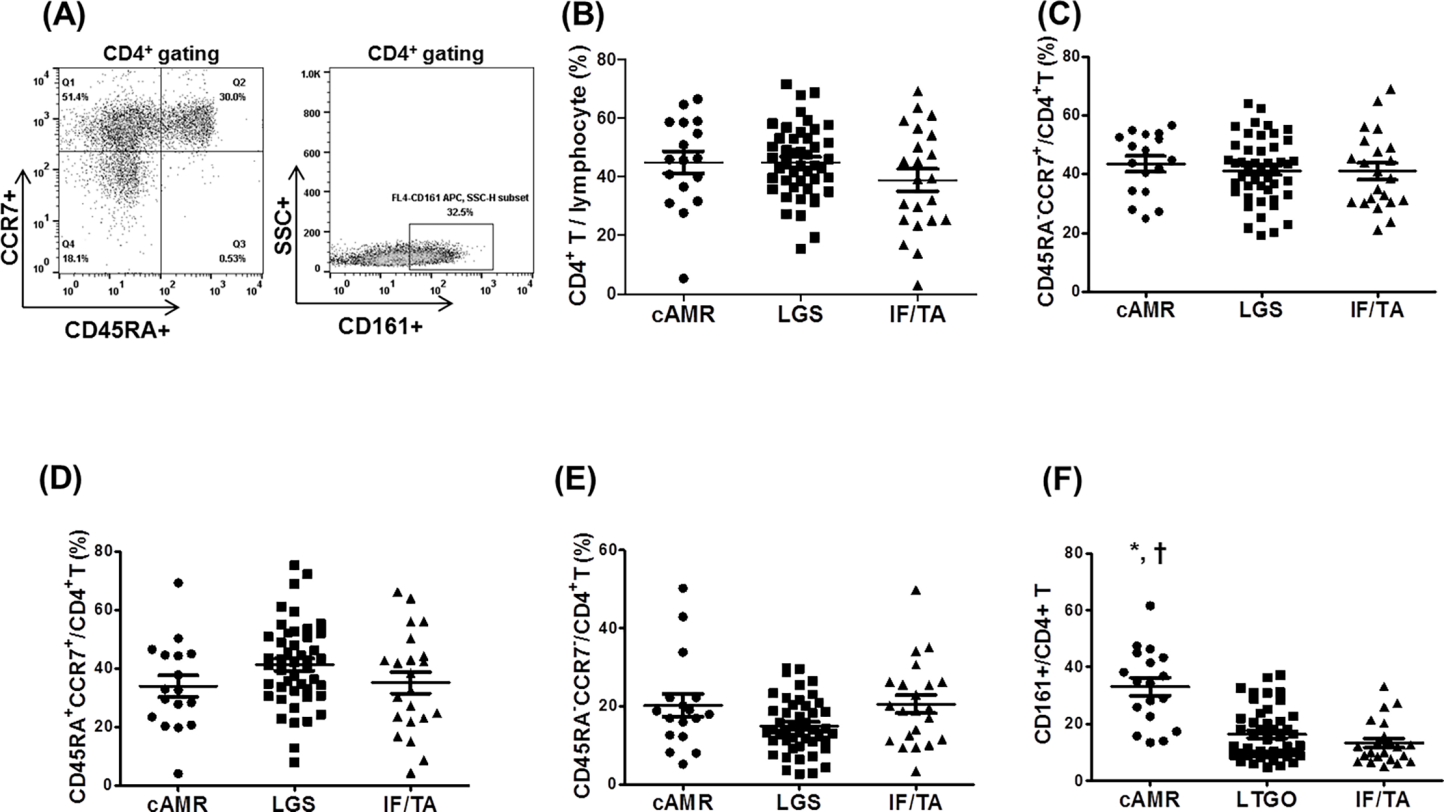
Comparison of CD4^+^ T cell subset among cAMR, IFTA, and LTGS groups. (A) PBMCs were stained with anti-CD4 PE-cy7, anti-CD45RA–FITC, and anti-CCR7 APC antibodies. CD4^+^ T cells were gated for further analysis. (B-F) Proportion (%) of (B) CD4^+^ T cells/lymphocytes, (C) TCM/CD4^+^ T (CD45RA–CCR7^+^/CD4^+^ T cells), (D) T naïve/CD4^+^ T (CD45RA^+^CCR7^+^/CD4^+^ T cells), (E) TEM/CD4^+^ T (CD45RA^–^CCR7^–^/CD4^+^ T cells), (F) CD161^+^/CD4^+^ T cells in each patient group. *p<0.05 for LTGS, †*p*<0.05 for IF/TA. Abbreviations; cAMR, chronic antibody-mediated rejection; LTGS; long-term graft survival; IF/TA, interstitial fibrosis/tubular atrophy.

The amount of proteinuria was significantly higher in the cAMR group than the other two groups and did not differ between LTGS and IF/TA groups. No difference was found in the gender, donor type, and HLA mismatch number among the three groups. In analysis of the CD4^+^ T cell population and its subsets, the proportion of CD4^+^ T lymphocytes (Fig 3B) among total lymphocytes, and the proportion of central memory (Fig 3C), naïve (Fig 3D), and effector memory (Fig 3E) CD4^+^ T cells among total CD4^+^ T cells by gating did not differ among the three groups. In contrast, the proportion of CD161^+^/CD4^+^ T cells was significantly higher in the cAMR group than the other two groups (*p*<0.01 for each) (Fig 3F). In the Fig 3, the proportion of CD161^+^CD4^+^ T cells showed a significant increase in KTRs with cAMR compared with KTRs with LTGS or non-specific IF/TA.

### Comparison of CD161^+^ cell infiltration in allograft tissue of cAMR and IF/TA groups

**Fig 4:**
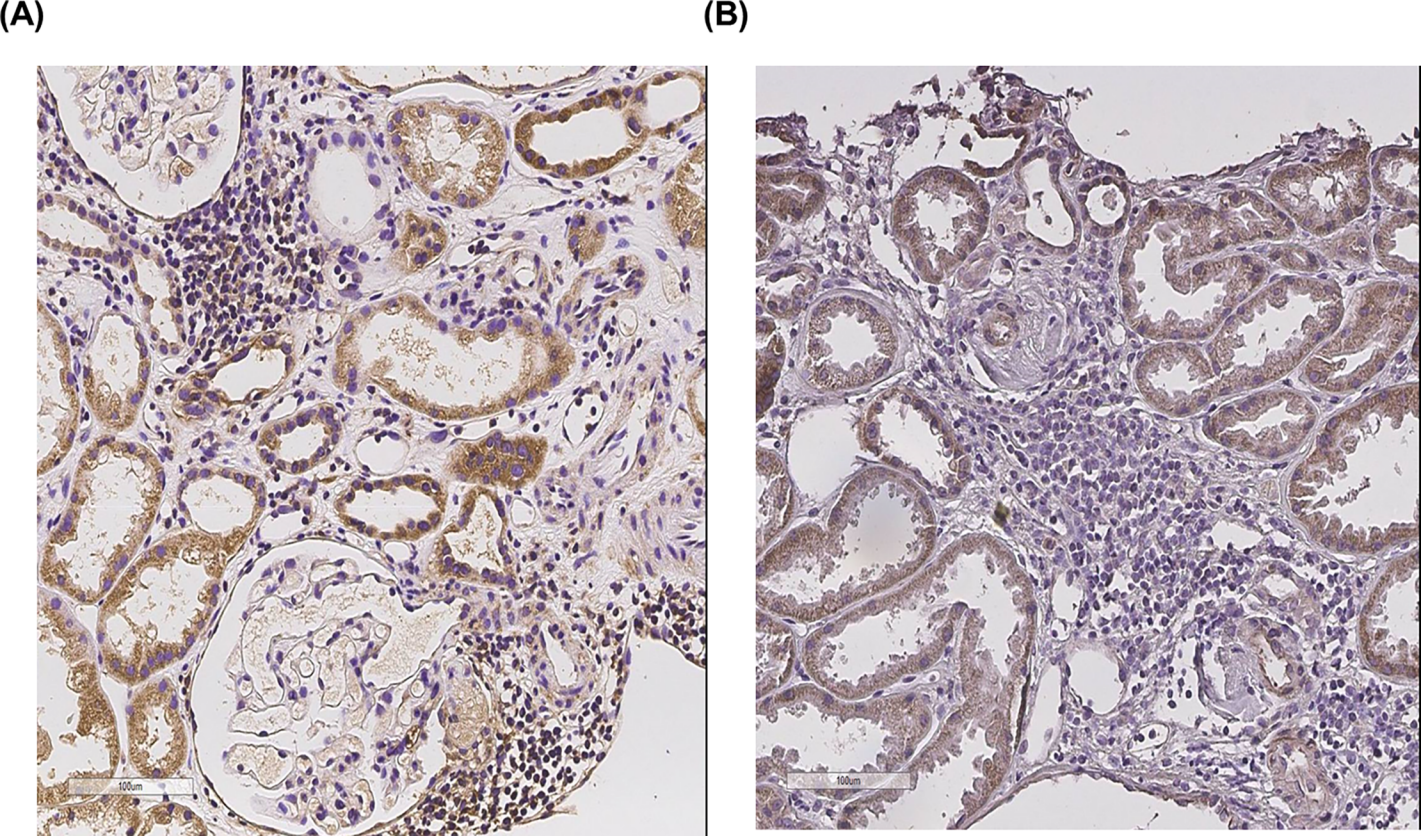
Comparison of CD161^+^ cell infiltration between cAMR group and IF/TA group. Representative staining of CD161^+^ cells in renal allograft tissue of (A) cAMR and (B) IF/TA groups. CD161^+^ cells were found mostly within interstitial lymphocyte infiltration (A, B: Original magnification ×400). cAMR, chronic antibody-mediated rejection; IF/TA, interstitial fibrosis/tubular atrophy.

Fig 4 shows representative staining of CD161^+^ cells in renal allograft tissue from the cAMR group (Fig 4A) and IF/TA group (Fig 4B). Positive CD161 staining was mostly found within interstitial lymphocyte infiltration in the cAMR group, but was rarely detected in the IF/TA group. The average number of infiltrating CD161^+^ cells was significantly higher in the cAMR group (20.6±14.5 cells/mm^2^) than in the IF/TA group (3.3±2.6 cells/mm^2^) (*p*<0.05). Fig 4 show that CD161^+^ cells was significantly higher in the cAMR group than in the IF/TA group.

### The effect of CD161^+^ T cells on inflammatory cytokine production in cultured human renal proximal tubular epithelial cells

**Fig 5:**
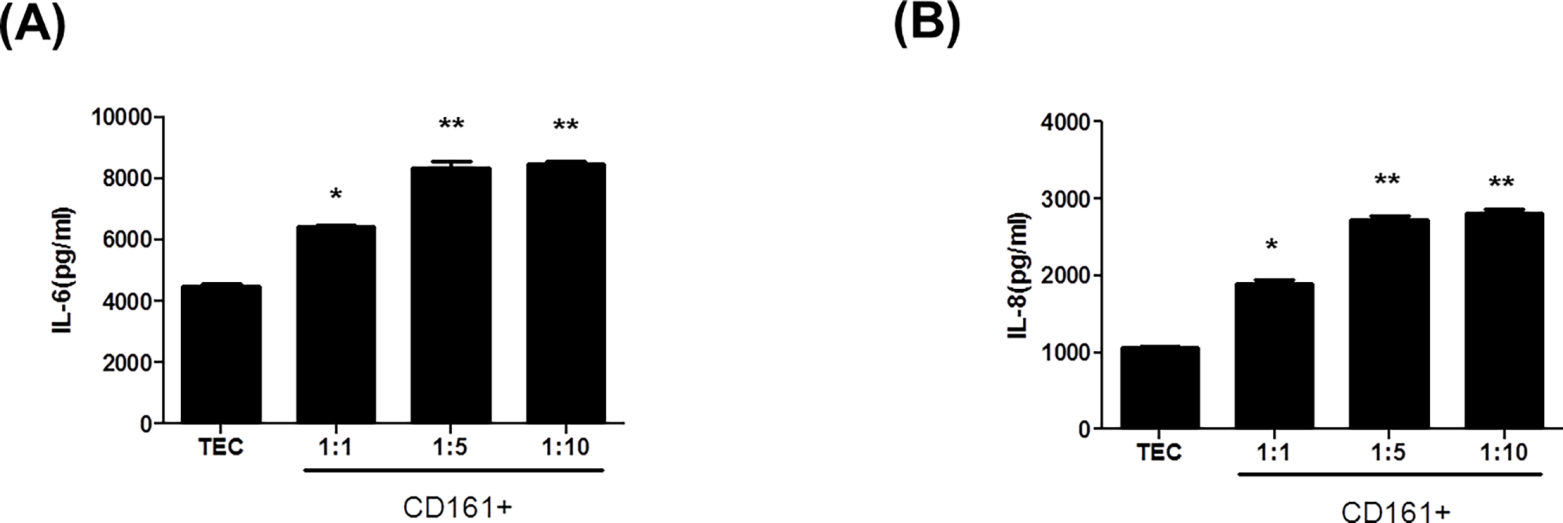
CD161^+^ T cells induced inflammation in human renal tubular epithelial cells. (A) IL-6 and (B) IL-8 productions by HRPTEpiC that were co-cultured for 48 or 72 hours with sorted CD161^+^ T cells. Treatment with sorted CD161^+^T cells increased the production of IL-6 and IL-8 by HRPTEpiC. *p<0.05 vs. TEC. Values are expressed as the mean and SD of triplicate cultures. HRPTEpiC (TEC), human renal proximal tubular epithelial cells.

Co-culture of HRPTEpiC with isolated CD161^+^ T cells (1:10 ratio) significantly increased the production of IL-6 (8,462±185 pg/ml and IL-8 (2,809±98 pg/ml) compared to HRPTEpiC alone (IL-6, 4,477±194 pg/ml; IL-8, 1,057±28 pg/ml) (*p<0.05, ** *p*<0.01 vs. HRPTEpiC alone) (Fig 5A and 5B). Therefore, addition of CD161^+^ T cells increased the production of these inflammatory cytokines by HRPTEpiC. Fig 5 show that the CD161^+^ T cells induced inflammation in human renal tubular epithelial cells.

## Discussion

In this study, we found that CD161^+^CD4^+^ T cells showed a significant correlation with IL-17 producing CD4^+^ T cells (Th17 cells) in *ex vivo* and *in vitro* studies. Furthermore, microarray analysis showed that transcripts associated with the Th17 pathway were upregulated in CD161^+^ T cells. The proportion of CD161^+^CD4^+^ T cells showed a significant increase in KTRs with cAMR compared with KTRs with LTGS or non-specific IF/TA. This finding suggests that an increase in CD161^+^ T cells may have a significant role in the progression of cAMR in KTRs.

First, we confirmed whether CD161^+^ T cells indicate activated Th17 cells. In this study, the proportion of CD161^+^ T cells showed a significant correlation with the proportion of Th17 cells, and CD161^+^ T cells showed a significant increase under Th17 activation conditions in an *in vitro* study. Those findings suggest that CD161^+^ T cells may share the characteristics of Th17 cells. In microarray analysis of sorted CD161^+^ T and CD161^-^ T cells, transcripts associated with activation of the Th17 pathway such as *IL18RAP*, *IL-18R1*, *IL23R*, *IL12RB2*, *RORC*, *TBX21*, and *EOMES* were upregulated in CD161^+^ T cells. Engagement of IL-18R alpha is required for the generation of pathogenic IL-17-producing T helper cells [19] and *IL18R* was one of the defining components of the leading edge gene set associated with CD161 expression. This receptor is composed of two subunits: IL18Ra and IL18RAP [20]. Il23R and RORC were reported to be TH17 lineage-specific genes [21]. Furthermore, Th17 cell plasticity is dependent on both STAT4 and Tbx21/T-bet, and it is important to determine the epigenetic profiles of genes such as Il12rb2 (whose expression is required for IL-12/Stat4 signaling) and Tbx21 [22]. Both T-bet and Eomes play direct roles in IFN-*γ* production and Th1 development, and T-bet plays a critical role in the choice between Th1 and Th17 development. [23].

Next, we examined whether CD161^+^CD4^+^ T cells show clinical significance in kidney transplant recipients. We previously reported that an increase in Th17 cells was associated with the progression of chronic allograft dysfunction in kidney transplant recipients [11]. In that study, we defined the CAD group as KTRs who were at least 2 years post-KT and showed not only morphological evidence of the presence of IF/TA but also functional deterioration, usually defined as estimated glomerular filtration rate (eGFR) <40 mL/min/1.73 m^2^ [11, 24, 25]. However, the most important limitation of that study was that the patient population was very heterogeneous and might not reflect cAMR, which is the most important cause of late allograft failure from non-specific IF/TA or chronic allograft nephropathy. Therefore, in this study we divided the patients into cAMR and IF/TA according to Banff classification [26]. As a control group, we included the LTGS group from multiple transplant centers; these patients were at least 10 years post-transplantation and showed MDRD eGFR > 50 mL/min/1.73 m^2^ [27].

We investigated the T cell phenotype in each group using multi-color FACS and compared the results. Interestingly, the most prominent finding was the significant increase in the proportion of CD161^+^CD4^+^ T cells in the cAMR group compared with IF/TA or LTGS control groups. Previously, we reported that the uremic condition induced by renal dysfunction can be associated with activation of the Th17 pathway [28]. However, in comparison between the cAMR and CAD groups, CD161^+^ T cells were significantly increased in the cAMR group compared with the IF/TA group although renal function was similar between two groups. The above findings suggest that the immunologic process rather than renal dysfunction may be primarily involved in the increase in CD161^+^CD4^+^ T cells in the cAMR group. In addition, we examined the naïve and memory T cells by staining peripheral blood T cells with antibodies to CD45RA and CCR7. The entire cohort of T_naïve_, T_CM,_ and T_EM_ cells did not show any difference between the CAD and LTS groups. T_naïve_ cells usually represent the immune cell pool that can be recruited in active infectious conditions; in contrast, T_EM_ cells represent actively differentiated immune cells [29, 30]. Hence, these results suggest that the immune cell pool and also non-specific activated T cells did not differ among these three groups.

Previously, we showed that Th17 infiltration is associated with more severe allograft tissue injury and poor allograft outcomes [9, 10]. In addition, we also found that the Th17 pathway was activated in the chronic antibody-mediated rejection group [11]. Based on those findings and the significant correlation between CD161^+^ T cells and the Th17 pathway in this study, we thought that a significant portion of infiltrating cells in allograft tissue from cAMR would express CD161. Finally, we found that the infiltrating number of CD161^+^ cells was significantly increased in the cAMR group compared with the IF/TA group, which is consistent with the difference in the level of CD161^+^ cells in peripheral blood.

Lastly, we tested the effect of CD161^+^CD4^+^ T cells on renal tubular epithelial cells *in vitro* to prove that these cells caused direct damage to renal tubular epithelial cells. We selected IL-6 and IL-8 secretion by renal tubular epithelial cells as markers of damage in renal tubular epithelial cells [31, 32]. We found a dose-dependent increase in these damage markers in HRPTEpiC co-cultured with CD161^+^ T cells. This result is fully consistent with previous reports suggesting that chronic renal tubular injury is caused by Th17-associated cytokines, and may partially explain the development of chronic allograft dysfunction induced by IL-17 [33, 34].

This study may have some limitations. For example, we did not perform allograft biopsy in the LTS group as in the previous study; therefore it is possible that chronic damage may exist in allograft tissue from patients in this group. However, considering the contrasting clinical status between LTGS and the other two groups we did not expect significant overlap among those groups, and even if chronic change is present in the allograft tissue of the LTGS group it is likely to be far less severe compared with the other groups. Second, as this study was performed as a cross-sectional design we did not show the impact of the CD161^+^CD4^+^ T cells on future clinical outcome. Sequential monitoring in a prospective cohort may be required to clarify this issue.

In conclusion, we found that CD161^+^CD4^+^ T cells showed a significant association with activation of the Th17 pathway in *in vitro* and *ex vivo* analyses. In addition, the proportion of CD161^+^CD4^+^ T cells was significantly increased in KTRs with cAMR, not only in peripheral blood but also in allograft tissue infiltrations. The results of this study indicate that monitoring of CD161^+^CD4^+^ T cells may be useful to detect the progression of cAMR in patients with chronic allograft dysfunction.

## Supporting information

S1 Fig GO analysis pathway annotation of CD161^+^ T Cells in comparison with CD161^-^ T Cells. (A) Biological process analysis of CD161^+^ T Cells in comparison with CD161^-^T Cells.
(B) Cellular component analysis of CD161^+^ T Cells in comparison with CD161^-^T Cells.
(C) Molecular function analysis of CD161^+^ T Cells in comparison with CD161^-^T Cells.
(DOCX)

S1 Table Up-regulated genes in CD161^+^ T cells compared with CD161^-^ T cells. (DOCX)

S2 Table Down-regulated genes in CD161^+^ T cells compared with CD161^-^ T cells. (DOCX)

## References

[1] Park H, Li Z, Yang XO, Chang SH, Nurieva R, Wang YH, et al A distinct lineage of CD4 T cells regulates tissue inflammation by producing interleukin 17. Nat Immunol. 2005;6(11):1133–41. Epub 2005/10/04. doi: ni1261 [pii] 10.1038/ni1261 ; PubMed Central PMCID: PMC1618871. 16200068 PMC1618871

[2] Steinman L. A brief history of T(H)17, the first major revision in the T(H)1/T(H)2 hypothesis of T cell-mediated tissue damage. Nat Med. 2007;13(2):139–45. Epub 2007/02/10. doi: nm1551 [pii] 10.1038/nm1551 . 17290272

[3] Loong CC, Lin CY, Lui WY. Expression of interleukin-17 as a predictive parameter in acute renal allograft rejection. Transplantation proceedings. 2000;32(7):1773–. 11119928 10.1016/s0041-1345(00)01382-8

[4] Hsieh HG, Loong CC, Lui WY, Chen A, Lin CY. IL-17 expression as a possible predictive parameter for subclinical renal allograft rejection. Transpl Int. 2001;14(5):287–98. Epub 2001/11/03. . 11692212 10.1007/s001470100344

[5] Mitchell P, Afzali B, Lombardi G, Lechler RI. The T helper 17-regulatory T cell axis in transplant rejection and tolerance. Curr Opin Organ Transplant. 2009;14(4):326–31. Epub 2009/05/19. 10.1097/MOT.0b013e32832ce88e . 19448538

[6] Crispim JC, Grespan R, Martelli-Palomino G, Rassi DM, Costa RS, Saber LT, et al Interleukin-17 and kidney allograft outcome. Transplant Proc. 2009;41(5):1562–4. Epub 2009/06/24. doi: S0041-1345(09)00260-7 [pii] 10.1016/j.transproceed.2009.01.092 . 19545679

[7] San Segundo D, Lopez-Hoyos M, Fernandez-Fresnedo G, Benito MJ, Ruiz JC, Benito A, et al T(H)17 versus Treg cells in renal transplant candidates: effect of a previous transplant. Transplant Proc. 2008;40(9):2885–8. Epub 2008/11/18. doi: S0041-1345(08)01335-3 [pii] 10.1016/j.transproceed.2008.09.043 . 19010136

[8] Chung BH, Kim KW, Kim BM, Piao SG, Lim SW, Choi BS, et al Dysregulation of Th17 cells during the early post-transplant period in patients under calcineurin inhibitor based immunosuppression. PLoS One. 2012;7(7):e42011 Epub 2012/08/01. 10.1371/journal.pone.0042011 PONE-D-11-08927 [pii]. ; PubMed Central PMCID: PMC3405048. 22848688 PMC3405048

[9] Chung BH, OH HJ, Piao SG, Sun IO, Kang SH, Choi SR, et al Higher infiltration by Th17 cells compared with regulatory T cells is associated with severe acute T-cell-mediated rejection. Exp Mol Med. 2011;43(11):630–7. Epub 2011 Aug 24. 10.3858/emm.2011.43.11.071 21865860 PMC3249589

[10] Chung BH, Oh HJ, Piao SG, Hwang HS, Sun IO, Choi SR, et al Clinical significance of the ratio between FOXP3 positive regulatory T cell and interleukin-17 secreting cell in renal allograft biopsies with acute T-cell-mediated rejection. Immunology. 2012;136(3):344–51. Epub 2012/03/27. 10.1111/j.1365-2567.2012.03588.x ; PubMed Central PMCID: PMC3385034. 22444300 PMC3385034

[11] Chung BH, Kim KW, Kim BM, Doh KC, Cho ML, Yang CW. Increase of Th17 Cell Phenotype in Kidney Transplant Recipients with Chronic Allograft Dysfunction. PLoS One. 2015;10(12):e0145258 10.1371/journal.pone.0145258 ; PubMed Central PMCID: PMCPMC4696852. 26717145 PMC4696852

[12] Cosmi L, De Palma R, Santarlasci V, Maggi L, Capone M, Frosali F, et al Human interleukin 17-producing cells originate from a CD161+CD4+ T cell precursor. J Exp Med. 2008;205(8):1903–16. 10.1084/jem.20080397 ; PubMed Central PMCID: PMC2525581. 18663128 PMC2525581

[13] Crome SQ, Wang AY, Levings MK. Translational mini-review series on Th17 cells: function and regulation of human T helper 17 cells in health and disease. Clin Exp Immunol. 2010;159(2):109–19. 10.1111/j.1365-2249.2009.04037.x ; PubMed Central PMCID: PMCPMC2810379. 19912252 PMC2810379

[14] Annunziato F, Cosmi L, Liotta F, Maggi E, Romagnani S. Defining the human T helper 17 cell phenotype. Trends Immunol. 2012;33(10):505–12. 10.1016/j.it.2012.05.004 . 22682163

[15] Lee SE, Lim JY, Yoon JH, Shin SH, Cho BS, Eom KS, et al CD161(+) T cells as predictive markers for acute graft-versus-host disease. Biol Blood Marrow Transplant. 2015;21(3):421–8. 10.1016/j.bbmt.2014.12.021 . 25543092

[16] van Besouw NM, Caliskan K, Peeters AM, Klepper M, Dieterich M, Maat LP, et al Interleukin-17-producing CD4(+) cells home to the graft early after human heart transplantation. J Heart Lung Transplant. 2015;34(7):933–40. 10.1016/j.healun.2014.12.013 . 25682556

[17] Solez K, Colvin RB, Racusen LC, Haas M, Sis B, Mengel M, et al Banff 07 classification of renal allograft pathology: updates and future directions. Am J Transplant. 2008;8(4):753–60. Epub 2008/02/26. 10.1111/j.1600-6143.2008.02159.x AJT2159 [pii]. . 18294345

[18] Alvarez-Lara MA, Carracedo J, Ramirez R, Martin-Malo A, Rodriguez M, Madueno JA, et al The imbalance in the ratio of Th1 and Th2 helper lymphocytes in uraemia is mediated by an increased apoptosis of Th1 subset. Nephrol Dial Transplant. 2004;19(12):3084–90. Epub 2004/12/03. doi: 19/12/3084 [pii] 10.1093/ndt/gfh382 . 15574999

[19] Gutcher I, Urich E, Wolter K, Prinz M, Becher B. Interleukin 18-independent engagement of interleukin 18 receptor-alpha is required for autoimmune inflammation. Nat Immunol. 2006;7(9):946–53. 10.1038/ni1377 . 16906165

[20] Fergusson JR, Smith KE, Fleming VM, Rajoriya N, Newell EW, Simmons R, et al CD161 defines a transcriptional and functional phenotype across distinct human T cell lineages. Cell Rep. 2014;9(3):1075–88. 10.1016/j.celrep.2014.09.045 ; PubMed Central PMCID: PMCPMC4250839. 25437561 PMC4250839

[21] Durant L, Watford WT, Ramos HL, Laurence A, Vahedi G, Wei L, et al Diverse targets of the transcription factor STAT3 contribute to T cell pathogenicity and homeostasis. Immunity. 2010;32(5):605–15. 10.1016/j.immuni.2010.05.003 ; PubMed Central PMCID: PMCPMC3148263. 20493732 PMC3148263

[22] Mukasa R, Balasubramani A, Lee YK, Whitley SK, Weaver BT, Shibata Y, et al Epigenetic instability of cytokine and transcription factor gene loci underlies plasticity of the T helper 17 cell lineage. Immunity. 2010;32(5):616–27. 10.1016/j.immuni.2010.04.016 ; PubMed Central PMCID: PMCPMC3129685. 20471290 PMC3129685

[23] Yang Y, Xu J, Niu Y, Bromberg JS, Ding Y. T-bet and eomesodermin play critical roles in directing T cell differentiation to Th1 versus Th17. J Immunol. 2008;181(12):8700–10. ; PubMed Central PMCID: PMCPMC2834216. 19050290 10.4049/jimmunol.181.12.8700 PMC2834216

[24] KDIGO clinical practice guideline for the care of kidney transplant recipients. Am J Transplant. 2009;9((Suppl 3)):S1–S157. 10.1111/j.1600-6143.2009.02834.x 19845597

[25] Maluf DG, Dumur CI, Suh JL, Lee JK, Cathro HP, King AL, et al Evaluation of molecular profiles in calcineurin inhibitor toxicity post-kidney transplant: input to chronic allograft dysfunction. Am J Transplant. 2014;14(5):1152–63. Epub 2014/04/05. 10.1111/ajt.12696 . 24698514 PMC4377109

[26] Haas M, Sis B, Racusen LC, Solez K, Glotz D, Colvin RB, et al Banff 2013 meeting report: inclusion of c4d-negative antibody-mediated rejection and antibody-associated arterial lesions. Am J Transplant. 2014;14(2):272–83. 10.1111/ajt.12590 . 24472190

[27] Seo JW, Moon H, Kim SY, Moon JY, Jeong KH, Lee YH, et al Both absolute and relative quantification of urinary mRNA are useful for non-invasive diagnosis of acute kidney allograft rejection. PLoS One. 2017;12(6):e0180045 10.1371/journal.pone.0180045 ; PubMed Central PMCID: PMCPMC5487057. 28654700 PMC5487057

[28] Chung BH, Kim KW, Sun IO, Choi SR, Park HS, Jeon EJ, et al Increased interleukin-17 producing effector memory T cells in the end-stage renal disease patients. Immunol Lett. 2011;141(2):181–9. Epub 2011 Oct 8. 10.1016/j.imlet.2011.10.002 22004873

[29] Gupta S, Bi R, Su K, Yel L, Chiplunkar S, Gollapudi S. Characterization of naive, memory and effector CD8+ T cells: effect of age. Exp Gerontol. 2004;39(4):545–50. Epub 2004/03/31. 10.1016/j.exger.2003.08.013 S053155650400021X [pii]. . 15050289

[30] Sallusto F, Lenig D, Forster R, Lipp M, Lanzavecchia A. Two subsets of memory T lymphocytes with distinct homing potentials and effector functions. Nature. 1999;401(6754):708–12. Epub 1999/10/28. 10.1038/44385 . 10537110

[31] Gore-Hyer E, Shegogue D, Markiewicz M, Lo S, Hazen-Martin D, Greene EL, et al TGF-beta and CTGF have overlapping and distinct fibrogenic effects on human renal cells. Am J Physiol Renal Physiol. 2002;283(4):F707–16. Epub 2002/09/10. 10.1152/ajprenal.00007.2002 . 12217862

[32] LeBleu VS, Taduri G, O'Connell J, Teng Y, Cooke VG, Woda C, et al Origin and function of myofibroblasts in kidney fibrosis. Nat Med. 2013;19(8):1047–53. Epub 2013/07/03. 10.1038/nm.3218 nm.3218 [pii]. ; PubMed Central PMCID: PMC4067127. 23817022 PMC4067127

[33] Van Kooten C, Boonstra JG, Paape ME, Fossiez F, Banchereau J, Lebecque S, et al Interleukin-17 activates human renal epithelial cells in vitro and is expressed during renal allograft rejection. J Am Soc Nephrol. 1998;9(8):1526–34. Epub 1998/08/11. . 9697677 10.1681/ASN.V981526

[34] Woltman AM, de Haij S, Boonstra JG, Gobin SJ, Daha MR, van Kooten C. Interleukin-17 and CD40-ligand synergistically enhance cytokine and chemokine production by renal epithelial cells. J Am Soc Nephrol. 2000;11(11):2044–55. Epub 2000/10/29. . 11053480 10.1681/ASN.V11112044

