# Supplementary material for: Clinical significance of CD161+CD4+ T cells in the development of chronic antibody-mediated rejection in kidney transplant recipients
Source: PLoS One. 2018 Jul 16;13(7):e0200631. doi: 10.1371/journal.pone.0200631 (PMC6047803; doi:10.1371/journal.pone.0200631)

**S1 Fig. GO analysis pathway annotation of CD161^+^ T Cells in comparison with CD161^-^T Cells.**

(A) Biological process analysis of CD161^+^ T Cells in comparison with CD161^-^T Cells.

(B) Cellular component analysis of CD161^+^ T Cells in comparison with CD161^-^T Cells.

(C) Molecular function analysis of CD161^+^ T Cells in comparison with CD161^-^T Cells.


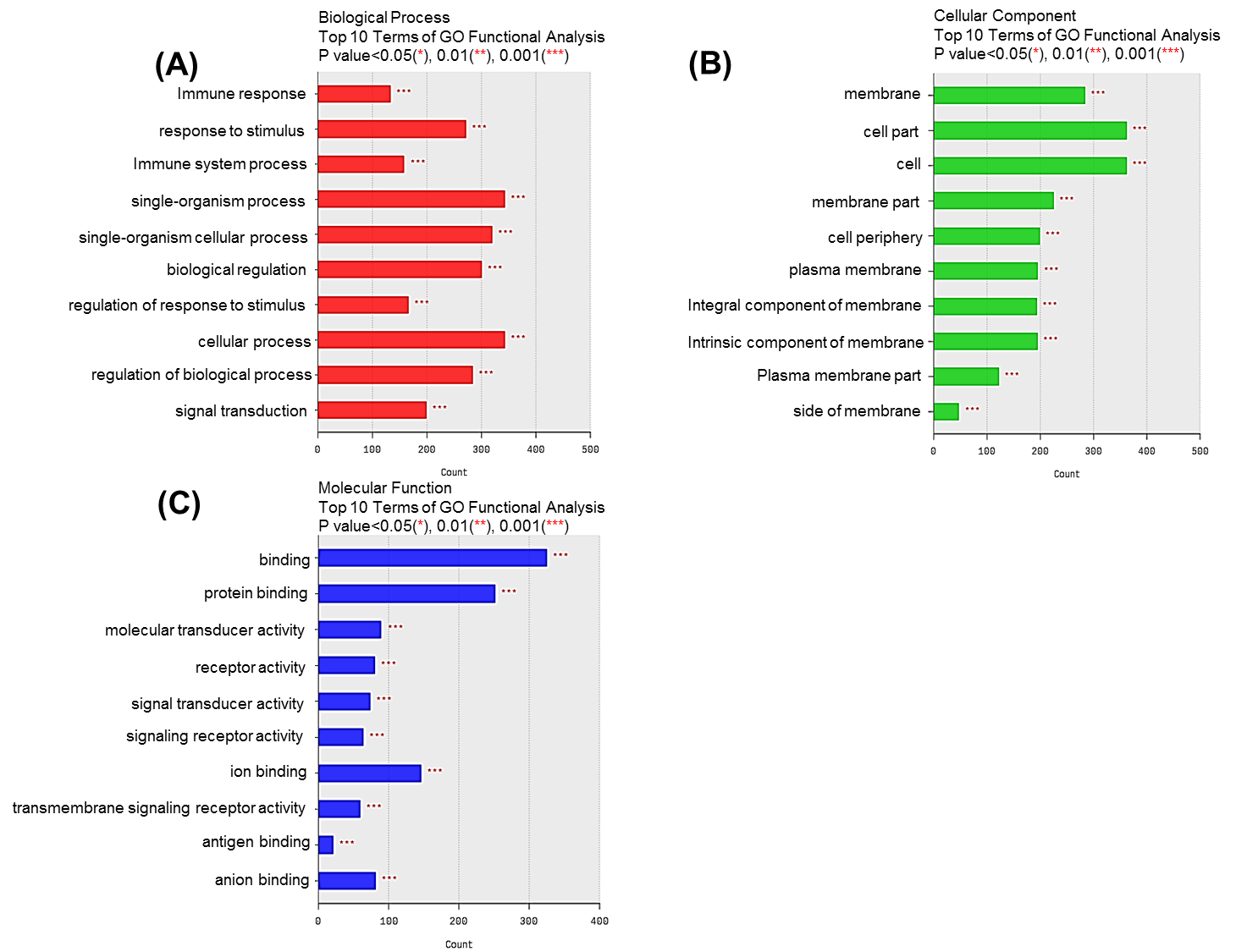

Supplement: S1 Fig — (A) Biological process analysis of CD161+ T Cells in comparison with CD161-T Cells. (B) Cellular component analysis of CD161+ T Cells in comparison with CD161-T Cells. (C) Molecular function analysis of CD161+ T Cells in comparison with CD161-T Cells. (DOCX) [file pone.0200631.s001.docx]
