# Supplementary material for: Clinical significance of CD161+CD4+ T cells in the development of chronic antibody-mediated rejection in kidney transplant recipients
Source: PLoS One. 2018 Jul 16;13(7):e0200631. doi: 10.1371/journal.pone.0200631 (PMC6047803; doi:10.1371/journal.pone.0200631)
Supplement: S1 Table — (DOCX) [file pone.0200631.s002.docx]

**S1 Table. Up-regulated genes in CD161^+^ T cells compared with CD161^-^ T cells.**

| **Gene Accession** | **Gene_Symbol** | **Gene Description** | **FC** |
| --- | --- | --- | --- |
| NM_002258 | KLRB1 | killer cell lectin-like receptor subfamily B, member 1 | 72.6 |
| NM_003853 | IL18RAP | interleukin 18 receptor accessory protein | 16.8 |
| NM_053282 | SH2D1B | SH2 domain containing 1B | 14.0 |
| OTTHUMT00000410935 | TRDJ3 | T cell receptor delta joining 3 | 13.4 |
| OTTHUMT00000410933 | TRDJ4 | T cell receptor delta joining 4 | 10.4 |
| NM_001291822 | KLRF1 | killer cell lectin-like receptor subfamily F, member 1 | 8.5 |
| NM_003783 | B3GALT2 | UDP-Gal:betaGlcNAc beta 1,3-galactosyltransferase 2 | 8.3 |
| NR_004386 | RNU105B | RNA, U105B small nucleolar | 8.0 |
| OTTHUMT00000410673 | TRDJ1 | T cell receptor delta joining 1 | 7.7 |
| NM_144701 | IL23R | interleukin 23 receptor | 7.5 |
| NM_001258214 | IL12RB2 | interleukin 12 receptor, beta 2 | 7.1 |
| BC072396 | TRGV9 | T cell receptor gamma variable 9 | 6.6 |
| NM_004775 | B4GALT6 | UDP-Gal:betaGlcNAc beta 1,4- galactosyltransferase, polypeptide 6 | 6.5 |
| OTTHUMT00000410936 | TRDC | T cell receptor delta constant | 5.9 |
| NM_001173514 | TYROBP | TYRO protein tyrosine kinase binding protein | 5.4 |
| OTTHUMT00000338835 | TRGJP1 | T cell receptor gamma joining P1 | 5.3 |
| NM_000615 | NCAM1 | neural cell adhesion molecule 1 | 5.0 |
| NM_180991 | SLCO4C1 | solute carrier organic anion transporter family, member 4C1 | 4.9 |
| NM_001304448 | KLRC1 | killer cell lectin-like receptor subfamily C, member 1 | 4.9 |
| NM_000887 | ITGAX | integrin alpha X | 4.9 |
| NM_014512 | KIR2DS1 | killer cell immunoglobulin-like receptor, two domains, short cytoplasmic tail, 1 | 4.8 |
| NM_015868 | KIR2DL3 | killer cell immunoglobulin-like receptor, two domains, long cytoplasmic tail, 3 | 4.8 |
| NM_015192 | PLCB1 | phospholipase C, beta 1 (phosphoinositide-specific) | 4.5 |
| NM_001136536 | DTHD1 | death domain containing 1 | 4.4 |
| NM_002288 | LAIR2 | leukocyte-associated immunoglobulin-like receptor 2 | 4.4 |
| OTTHUMT00000338828 | TRGJP2 | T cell receptor gamma joining P2 | 4.3 |
| NM_001135731 | ZMAT4 | zinc finger, matrin-type 4 | 4.3 |
| OTTHUMT00000351239 | TRBV6-4 | T cell receptor beta variable 6-4 | 4.3 |
| ENST00000598878 | METTL6 | methyltransferase like 6 | 4.2 |
| ENST00000610437 | KIR3DL3 | killer cell immunoglobulin-like receptor, three domains, long cytoplasmic tail, 3 | 4.2 |
| NM_001178015 | SLC4A10 | solute carrier family 4, sodium bicarbonate transporter, member 10 | 4.1 |
| OTTHUMT00000338399 | TRGV8 | T cell receptor gamma variable 8 | 4.1 |
| NM_001288973 | ADAM12 | ADAM metallopeptidase domain 12 | 4.0 |
| NM_000953 | PTGDR | prostaglandin D2 receptor (DP) | 4.0 |
| NM_015166 | MLC1 | megalencephalic leukoencephalopathy with subcortical cysts 1 | 4.0 |
| NM_012313 | KIR2DS3 | killer cell immunoglobulin-like receptor, two domains, short cytoplasmic tail, 3 | 4.0 |
| NM_001145457 | NCR1 | natural cytotoxicity triggering receptor 1 | 4.0 |
| NM_004106 | FCER1G | Fc fragment of IgE, high affinity I, receptor for; gamma polypeptide | 4.0 |
| NM_001277201 | SIGLEC7 | sialic acid binding Ig-like lectin 7 | 3.9 |
| NR_034135 | LINC00299 | long intergenic non-protein coding RNA 299 | 3.9 |
| NM_001018011 | ZBTB16 | zinc finger and BTB domain containing 16 | 3.8 |
| NM_014219 | KIR2DL2 | killer cell immunoglobulin-like receptor, two domains, long cytoplasmic tail, 2 | 3.8 |
| NM_001145466 | NCR3 | natural cytotoxicity triggering receptor 3 | 3.8 |
| NM_000632 | ITGAM | integrin, alpha M (complement component 3 receptor 3 subunit) | 3.7 |
| NM_001144903 | TM6SF1 | transmembrane 6 superfamily member 1 | 3.7 |
| BC072387 | TRGV9 | T cell receptor gamma variable 9 | 3.6 |
| NM_001004431 | METRNL | meteorin, glial cell differentiation regulator-like | 3.6 |
| OTTHUMT00000410934 | TRDJ2 | T cell receptor delta joining 2 | 3.6 |
| NM_000634 | CXCR1 | chemokine (C-X-C motif) receptor 1 | 3.6 |
| NR_003341 | SNORD116-27 | small nucleolar RNA, C/D box 116-27 | 3.6 |
| XR_913850 | LOC105369656 | uncharacterized LOC105369656 | 3.5 |
| NM_004131 | GZMB | granzyme B | 3.5 |
| NM_007053 | CD160 | CD160 molecule | 3.4 |
| NM_001282399 | IL18R1 | interleukin 18 receptor 1 | 3.4 |
| NM_001256841 | CD300A | CD300a molecule | 3.4 |
| NM_032782 | HAVCR2 | hepatitis A virus cellular receptor 2 | 3.4 |
| NM_001013742 | DGKK | diacylglycerol kinase, kappa | 3.3 |
| NM_002984 | CCL4 | chemokine (C-C motif) ligand 4 | 3.3 |
| NM_004669 | CLIC3 | chloride intracellular channel 3 | 3.3 |
| NM_001166215 | S1PR5 | sphingosine-1-phosphate receptor 5 | 3.2 |
| NM_002260 | KLRC2 | killer cell lectin-like receptor subfamily C, member 2 | 3.2 |
| NM_001242867 | KIR3DL2 | killer cell immunoglobulin-like receptor, three domains, long cytoplasmic tail, 2 | 3.2 |
| NM_003246 | THBS1 | thrombospondin 1 | 3.2 |
| NM_001775 | CD38 | CD38 molecule | 3.1 |
| ENST00000588679 | RNF165 | ring finger protein 165 | 3.1 |
| NM_001080416 | MYBL1 | v-myb avian myeloblastosis viral oncogene homolog-like 1 | 3.1 |
| NR_030618 | MIR873 | microRNA 873 | 3.1 |
| NM_014572 | LATS2 | large tumor suppressor kinase 2 | 3.1 |
| NM_001123041 | CCR2 | chemokine (C-C motif) receptor 2 | 3.0 |
| NM_000639 | FASLG | Fas ligand (TNF superfamily, member 6) | 3.0 |
| NM_017637 | BNC2 | basonuclin 2 | 3.0 |
| NM_004227 | CYTH3 | cytohesin 3 | 3.0 |
| NM_001253835 | IGFBP7 | insulin like growth factor binding protein 7 | 3.0 |
| XR_930865 | NCR3LG1 | natural killer cell cytotoxicity receptor 3 ligand 1 | 3.0 |
| NM_002261 | KLRC3 | killer cell lectin-like receptor subfamily C, member 3 | 3.0 |
| NM_001166663 | CD244 | CD244 molecule, natural killer cell receptor 2B4 | 2.9 |
| OTTHUMT00000338832 | TRGV10 | T cell receptor gamma variable 10 (non-functional) | 2.9 |
| XR_931192 | LOC105376626 | uncharacterized LOC105376626 | 2.9 |
| NM_001433 | ERN1 | endoplasmic reticulum to nucleus signaling 1 | 2.9 |
| NM_001128325 | SPON2 | spondin 2, extracellular matrix protein | 2.9 |
| NM_001282588 | SLAMF7 | SLAM family member 7 | 2.9 |
| NM_015103 | PLXND1 | plexin D1 | 2.9 |
| NM_001244871 | DAB2 | Dab, mitogen-responsive phosphoprotein, homolog 2 (Drosophila) | 2.9 |
| NM_001098484 | SLC4A4 | solute carrier family 4 (sodium bicarbonate cotransporter), member 4 | 2.9 |
| NM_004747 | DLG5 | discs, large homolog 5 (Drosophila) | 2.9 |
| NM_001039547 | GK5 | glycerol kinase 5 (putative) | 2.9 |
| NM_001114396 | KLRD1 | killer cell lectin-like receptor subfamily D, member 1 | 2.9 |
| NM_001080770 | KIR2DL4 | killer cell immunoglobulin-like receptor, two domains, long cytoplasmic tail, 4 | 2.9 |
| NM_001083116 | PRF1 | perforin 1 (pore forming protein) | 2.8 |
| NM_001303103 | TLE1 | transducin-like enhancer of split 1 (E(sp1) homolog, Drosophila) | 2.8 |
| NM_000878 | IL2RB | interleukin 2 receptor, beta | 2.8 |
| NM_182523 | CMC1 | C-x(9)-C motif containing 1 | 2.8 |
| NM_030806 | C1orf21 | chromosome 1 open reading frame 21 | 2.8 |
| NM_001083539 | KIR3DS1 | killer cell immunoglobulin-like receptor, three domains, short cytoplasmic tail, 1 | 2.8 |
| NM_001177506 | AOAH | acyloxyacyl hydrolase (neutrophil) | 2.8 |
| NM_001135685 | LTK | leukocyte receptor tyrosine kinase | 2.8 |
| NM_024837 | ATP8B4 | ATPase, class I, type 8B, member 4 | 2.8 |
| NR_026971 | A2M-AS1 | A2M antisense RNA 1 (head to head) | 2.8 |
| XR_937125 | LOC102724104 | uncharacterized LOC102724104 | 2.8 |
| NR_028092 | LPAL2 | lipoprotein, Lp(a)-like 2, pseudogene | 2.7 |
| NM_001291468 | CCL4L2 | chemokine (C-C motif) ligand 4-like 2 | 2.7 |
| NM_002104 | GZMK | granzyme K | 2.7 |
| NR_046764 | AOAH-IT1 | AOAH intronic transcript 1 | 2.7 |
| NM_001083914 | CTBP2 | C-terminal binding protein 2 | 2.7 |
| NM_006144 | GZMA | granzyme A | 2.7 |
| NM_006056 | NMUR1 | neuromedin U receptor 1 | 2.7 |
| NM_001031804 | MAF | v-maf avian musculoaponeurotic fibrosarcoma oncogene homolog | 2.6 |
| NM_001001523 | RORC | RAR-related orphan receptor C | 2.6 |
| NM_031950 | FGFBP2 | fibroblast growth factor binding protein 2 | 2.6 |
| NM_013351 | TBX21 | T-box 21 | 2.6 |
| NM_001162951 | SYTL2 | synaptotagmin-like 2 | 2.6 |
| NM_001017403 | LGR6 | leucine-rich repeat containing G protein-coupled receptor 6 | 2.6 |
| NM_001261835 | BZRAP1 | benzodiazepine receptor (peripheral) associated protein 1 | 2.6 |
| NM_005253 | FOSL2 | FOS-like antigen 2 | 2.6 |
| NM_003670 | BHLHE40 | basic helix-loop-helix family, member e40 | 2.5 |
| NM_004163 | RAB27B | RAB27B, member RAS oncogene family | 2.5 |
| NM_001270526 | STOM | stomatin | 2.5 |
| XR_430789 | LOC102724850 | uncharacterized LOC102724850 | 2.5 |
| NM_001061 | TBXAS1 | thromboxane A synthase 1 (platelet) | 2.5 |
| NM_003650 | CST7 | cystatin F (leukocystatin) | 2.5 |
| NM_001005505 | CACNA2D2 | calcium channel, voltage-dependent, alpha 2/delta subunit 2 | 2.5 |
| NM_024940 | DOCK5 | dedicator of cytokinesis 5 | 2.5 |
| NR_036466 | LINC00987 | long intergenic non-protein coding RNA 987 | 2.5 |
| NM_024896 | ERMP1 | endoplasmic reticulum metallopeptidase 1 | 2.5 |
| NR_034078 | LOC643733 | caspase 4, apoptosis-related cysteine peptidase pseudogene | 2.5 |
| NM_017931 | TTC38 | tetratricopeptide repeat domain 38 | 2.5 |
| NM_001146032 | FCHO2 | FCH domain only 2 | 2.5 |
| NM_001098802 | CEP78 | centrosomal protein 78kDa | 2.5 |
| NM_001159643 | MCTP2 | multiple C2 domains, transmembrane 2 | 2.4 |
| NM_001145770 | ADGRG1 | adhesion G protein-coupled receptor G1 | 2.4 |
| NM_001142339 | GNAL | guanine nucleotide binding protein (G protein), alpha activating activity polypeptide, olfactory type | 2.4 |
| NM_020531 | APMAP | adipocyte plasma membrane associated protein | 2.4 |
| NM_020133 | AGPAT4 | 1-acylglycerol-3-phosphate O-acyltransferase 4 | 2.4 |
| NR_026755 | CYP4F29P | cytochrome P450, family 4, subfamily F, polypeptide 29, pseudogene | 2.4 |
| NM_001037131 | AGAP1 | ArfGAP with GTPase domain, ankyrin repeat and PH domain 1 | 2.4 |
| NM_000416 | IFNGR1 | interferon gamma receptor 1 | 2.4 |
| NM_001335 | CTSW | cathepsin W | 2.4 |
| NM_000927 | ABCB1 | ATP binding cassette subfamily B member 1 | 2.4 |
| NR_038975 | MIR181A2HG | MIR181A2 host gene | 2.4 |
| BC039116 | TRGC2 | T cell receptor gamma constant 2 | 2.4 |
| NM_145648 | SLC15A4 | solute carrier family 15 (oligopeptide transporter), member 4 | 2.4 |
| NM_001131008 | PTPN12 | protein tyrosine phosphatase, non-receptor type 12 | 2.4 |
| NM_012335 | MYO1F | myosin IF | 2.4 |
| NM_003486 | SLC7A5 | solute carrier family 7 (amino acid transporter light chain, L system), member 5 | 2.4 |
| OTTHUMT00000487452 | CCL4L1 | chemokine (C-C motif) ligand 4-like 1 | 2.4 |
| XR_915959 | LOC105370660 | uncharacterized LOC105370660 | 2.4 |
| NM_001289823 | FURIN | furin (paired basic amino acid cleaving enzyme) | 2.3 |
| NM_006750 | SNTB2 | syntrophin, beta 2 (dystrophin-associated protein A1, 59kDa, basic component 2) | 2.3 |
| NM_144682 | SLFN13 | schlafen family member 13 | 2.3 |
| NM_001244134 | MAP3K8 | mitogen-activated protein kinase kinase kinase 8 | 2.3 |
| NM_001302758 | GNLY | granulysin | 2.3 |
| NM_014218 | KIR2DL1 | killer cell immunoglobulin-like receptor, two domains, long cytoplasmic tail, 1 | 2.3 |
| NR_047529 | SIGLEC17P | sialic acid binding Ig-like lectin 17, pseudogene | 2.3 |
| NM_005810 | KLRG1 | killer cell lectin-like receptor subfamily G, member 1 | 2.3 |
| NM_024310 | PLEKHF1 | pleckstrin homology domain containing, family F (with FYVE domain) member 1 | 2.3 |
| ENST00000394409 | PPP2R2B | protein phosphatase 2, regulatory subunit B, beta | 2.3 |
| NM_024329 | EFHD2 | EF-hand domain family member D2 | 2.3 |
| NM_004329 | BMPR1A | bone morphogenetic protein receptor type IA | 2.3 |
| OTTHUMT00000430715 | GOLGA8Q | golgin A8 family, member Q | 2.3 |
| AK021795 | MGC24103 | uncharacterized MGC24103 | 2.3 |
| NM_003608 | GPR65 | G protein-coupled receptor 65 | 2.3 |
| NM_001171971 | CDHR1 | cadherin-related family member 1 | 2.3 |
| NR_003326 | SNORD116-11 | small nucleolar RNA, C/D box 116-11 | 2.3 |
| NM_001606 | ABCA2 | ATP binding cassette subfamily A member 2 | 2.3 |
| NM_000271 | NPC1 | Niemann-Pick disease, type C1 | 2.3 |
| NM_001040078 | LGALS9C | lectin, galactoside-binding, soluble, 9C | 2.3 |
| NR_039930 | MIR4772 | microRNA 4772 | 2.2 |
| NM_001270934 | JAKMIP2 | janus kinase and microtubule interacting protein 2 | 2.2 |
| NM_001282472 | GOLGA8J | golgin A8 family, member J | 2.2 |
| NM_001243794 | CHST12 | carbohydrate (chondroitin 4) sulfotransferase 12 | 2.2 |
| NM_001143783 | FES | FES proto-oncogene, tyrosine kinase | 2.2 |
| NM_018690 | APOBR | apolipoprotein B receptor | 2.2 |
| NM_000302 | PLOD1 | procollagen-lysine, 2-oxoglutarate 5-dioxygenase 1 | 2.2 |
| NM_152353 | CLDND2 | claudin domain containing 2 | 2.2 |
| NM_001161441 | SH2D2A | SH2 domain containing 2A | 2.2 |
| NM_005907 | MAN1A1 | mannosidase, alpha, class 1A, member 1 | 2.2 |
| NM_001284194 | FUT11 | fucosyltransferase 11 (alpha (1,3) fucosyltransferase) | 2.2 |
| NM_000024 | ADRB2 | adrenoceptor beta 2, surface | 2.2 |
| NM_001289999 | NFIL3 | nuclear factor, interleukin 3 regulated | 2.2 |
| NM_001081573 | GAB3 | GRB2-associated binding protein 3 | 2.2 |
| NM_033071 | SYNE1 | spectrin repeat containing, nuclear envelope 1 | 2.2 |
| NR_109967 | LL22NC03-75H12.2 | uncharacterized LOC101927722 | 2.2 |
| NM_004711 | SYNGR1 | synaptogyrin 1 | 2.2 |
| NM_001282640 | SUSD1 | sushi domain containing 1 | 2.2 |
| NM_001202439 | NCR3LG1 | natural killer cell cytotoxicity receptor 3 ligand 1 | 2.2 |
| NM_014899 | RHOBTB3 | Rho-related BTB domain containing 3 | 2.2 |
| NM_001243198 | HIP1 | huntingtin interacting protein 1 | 2.2 |
| NM_001040100 | SPTSSB | serine palmitoyltransferase, small subunit B | 2.2 |
| NM_001143781 | FKBP11 | FK506 binding protein 11 | 2.2 |
| XR_923086 | LOC105373495 | uncharacterized LOC105373495 | 2.1 |
| NM_152501 | PYHIN1 | pyrin and HIN domain family, member 1 | 2.1 |
| NM_001304376 | ADGRG5 | adhesion G protein-coupled receptor G5 | 2.1 |
| NM_001161748 | LIM2 | lens intrinsic membrane protein 2 | 2.1 |
| NM_001171171 | CX3CR1 | chemokine (C-X3-C motif) receptor 1 | 2.1 |
| XR_920216 | LOC105372674 | uncharacterized LOC105372674 | 2.1 |
| NM_001144063 | OSBPL5 | oxysterol binding protein-like 5 | 2.1 |
| NM_001105244 | PTPRM | protein tyrosine phosphatase, receptor type, M | 2.1 |
| NM_004482 | GALNT3 | polypeptide N-acetylgalactosaminyltransferase 3 | 2.1 |
| OTTHUMT00000401873 | TRAV1-2 | T cell receptor alpha variable 1-2 | 2.1 |
| NM_001278182 | EOMES | eomesodermin | 2.1 |
| NM_001145088 | TBC1D31 | TBC1 domain family, member 31 | 2.1 |
| NM_006981 | NR4A3 | nuclear receptor subfamily 4, group A, member 3 | 2.1 |
| NM_001311313 | F2R | coagulation factor II (thrombin) receptor | 2.1 |
| NM_006264 | PTPN13 | protein tyrosine phosphatase, non-receptor type 13 (APO-1/CD95 (Fas)-associated phosphatase) | 2.1 |
| NM_001135095 | FNDC3B | fibronectin type III domain containing 3B | 2.1 |
| NM_001193380 | IL17RE | interleukin 17 receptor E | 2.1 |
| NM_001253908 | AKR1C3 | aldo-keto reductase family 1, member C3 | 2.1 |
| NR_026716 | KIR3DX1 | killer cell immunoglobulin-like receptor, three domains, X1 | 2.1 |
| NM_025185 | TANC2 | tetratricopeptide repeat, ankyrin repeat and coiled-coil containing 2 | 2.1 |
| NR_034033 | LINC00996 | long intergenic non-protein coding RNA 996 | 2.1 |
| NM_006417 | IFI44 | interferon-induced protein 44 | 2.1 |
| NM_005601 | NKG7 | natural killer cell granule protein 7 | 2.1 |
| NM_002664 | PLEK | pleckstrin | 2.1 |
| NM_001102592 | HENMT1 | HEN1 methyltransferase homolog 1 (Arabidopsis) | 2.1 |
| NM_152723 | CCDC89 | coiled-coil domain containing 89 | 2.1 |
| NM_006564 | CXCR6 | chemokine (C-X-C motif) receptor 6 | 2.1 |
| XR_241262 | LOC101929531 | uncharacterized LOC101929531 | 2.0 |
| NM_001195683 | TGFBR3 | transforming growth factor beta receptor III | 2.0 |
| NM_001030272 | ARNTL | aryl hydrocarbon receptor nuclear translocator-like | 2.0 |
| NM_152280 | SYT11 | synaptotagmin XI | 2.0 |
| NM_004776 | B4GALT5 | UDP-Gal:betaGlcNAc beta 1,4- galactosyltransferase, polypeptide 5 | 2.0 |
| XM_005276144 | LOC100996721 | protein FAM231D | 2.0 |
| NM_004580 | RAB27A | RAB27A, member RAS oncogene family | 2.0 |
| NM_001145933 | TKTL1 | transketolase-like 1 | 2.0 |
| NM_000956 | PTGER2 | prostaglandin E receptor 2 | 2.0 |
| NM_001243878 | FHL3 | four and a half LIM domains 3 | 2.0 |
| NM_001015002 | LLGL2 | lethal giant larvae homolog 2 (Drosophila) | 2.0 |
| NM_006111 | ACAA2 | acetyl-CoA acyltransferase 2 | 2.0 |
| AF018171 | YME1L1 | YME1-like 1 ATPase | 2.0 |
| NM_001291274 | HSH2D | hematopoietic SH2 domain containing | 2.0 |
| NM_001204406 | ALOX5AP | arachidonate 5-lipoxygenase-activating protein | 2.0 |
| NM_001042388 | PPP4R1 | protein phosphatase 4, regulatory subunit 1 | 2.0 |
| M13231 | TRGC2 | T cell receptor gamma constant 2 | 2.0 |
| BC072387 | TRGV9 | T cell receptor gamma variable 9 | 2.0 |
| NM_001160167 | PRR5L | proline rich 5 like | 2.0 |
| NM_001100398 | RAP1GAP2 | RAP1 GTPase activating protein 2 | 2.0 |
| NM_024330 | SLC27A3 | solute carrier family 27 (fatty acid transporter), member 3 | 2.0 |
| NM_001100164 | PHACTR2 | phosphatase and actin regulator 2 | 2.0 |
| NM_017983 | WIPI1 | WD repeat domain, phosphoinositide interacting 1 | 2.0 |
| NM_000153 | GALC | galactosylceramidase | 2.0 |
| NR_002977 | SNORA3B | small nucleolar RNA, H/ACA box 3B | 2.0 |
| NM_181791 | GPR141 | G protein-coupled receptor 141 | 2.0 |
| ENST00000628473 | ZEB2 | zinc finger E-box binding homeobox 2 | 2.0 |
| NM_001174166 | SLC16A6 | solute carrier family 16, member 6 | 2.0 |
| NM_001308147 | PLEKHG3 | pleckstrin homology domain containing, family G (with RhoGef domain) member 3 | 1.9 |
| NM_001195790 | SLFN12L | schlafen family member 12-like | 1.9 |
| NM_000954 | PTGDS | prostaglandin D2 synthase 21kDa (brain) | 1.9 |
| NM_002609 | PDGFRB | platelet-derived growth factor receptor, beta polypeptide | 1.9 |
| NR_121674 | ERICH6-AS1 | ERICH6 antisense RNA 1 | 1.9 |
| NM_004522 | KIF5C | kinesin family member 5C | 1.9 |
| NM_001178138 | TFDP2 | transcription factor Dp-2 (E2F dimerization partner 2) | 1.9 |
| NM_003807 | TNFSF14 | tumor necrosis factor (ligand) superfamily, member 14 | 1.9 |
| NM_004419 | DUSP5 | dual specificity phosphatase 5 | 1.9 |
| NM_000734 | CD247 | CD247 molecule | 1.9 |
| BC073897 | MXRA7 | matrix-remodelling associated 7 | 1.9 |
| NR_030376 | MIR646 | microRNA 646 | 1.9 |
| NM_152781 | HEATR9 | HEAT repeat containing 9 | 1.9 |
| XM_011519310 | MAP3K8 | mitogen-activated protein kinase kinase kinase 8 | 1.9 |
| NM_001080480 | MBOAT1 | membrane bound O-acyltransferase domain containing 1 | 1.9 |
| NM_001042729 | FGR | FGR proto-oncogene, Src family tyrosine kinase | 1.9 |
| NM_001199775 | CPD | carboxypeptidase D | 1.9 |
| NM_032233 | SETD3 | SET domain containing 3 | 1.9 |
| NM_001039948 | SGSM1 | small G protein signaling modulator 1 | 1.9 |
| NM_016626 | MEX3C | mex-3 RNA binding family member C | 1.9 |
| NM_001040624 | NCALD | neurocalcin delta | 1.9 |
| NM_001303618 | CD226 | CD226 molecule | 1.9 |
| AK128046 | LOC100128668 | uncharacterized LOC100128668 | 1.9 |
| NM_001083885 | DFNB31 | deafness, autosomal recessive 31 | 1.9 |
| NM_001145199 | C12orf75 | chromosome 12 open reading frame 75 | 1.9 |
| NM_001135608 | ARHGAP26 | Rho GTPase activating protein 26 | 1.9 |
| NM_001859 | SLC31A1 | solute carrier family 31 (copper transporter), member 1 | 1.9 |
| NM_013254 | TBK1 | TANK-binding kinase 1 | 1.8 |
| NM_013313 | YPEL1 | yippee like 1 | 1.8 |
| NM_004924 | ACTN4 | actinin, alpha 4 | 1.8 |
| NM_001114173 | CTSC | cathepsin C | 1.8 |
| NM_052966 | FAM129A | family with sequence similarity 129, member A | 1.8 |
| ENST00000482608 | C8orf46 | chromosome 8 open reading frame 46 | 1.8 |
| NM_001570 | IRAK2 | interleukin 1 receptor associated kinase 2 | 1.8 |
| NM_015180 | SYNE2 | spectrin repeat containing, nuclear envelope 2 | 1.8 |
| NM_003175 | XCL2 | chemokine (C motif) ligand 2 | 1.8 |
| NM_001004310 | FCRL6 | Fc receptor-like 6 | 1.8 |
| NM_002378 | MATK | megakaryocyte-associated tyrosine kinase | 1.8 |
| NM_001006636 | GTDC1 | glycosyltransferase like domain containing 1 | 1.8 |
| NM_001178055 | PARP8 | poly(ADP-ribose) polymerase family member 8 | 1.8 |
| AK125248 | LOC728323 | uncharacterized LOC728323 | 1.8 |
| XR_917210 | LOC101929787 | uncharacterized LOC101929787 | 1.8 |
| NM_020733 | HEG1 | heart development protein with EGF-like domains 1 | 1.8 |
| NM_000876 | IGF2R | insulin-like growth factor 2 receptor | 1.8 |
| NM_001001396 | ATP2B4 | ATPase, Ca++ transporting, plasma membrane 4 | 1.8 |
| NR_036225 | MIR4267 | microRNA 4267 | 1.8 |
| NM_015175 | NBEAL2 | neurobeachin like 2 | 1.8 |
| NM_001316676 | PTPRE | protein tyrosine phosphatase, receptor type, E | 1.8 |
| NM_003659 | AGPS | alkylglycerone phosphate synthase | 1.8 |
| NM_014718 | CLSTN3 | calsyntenin 3 | 1.8 |
| NM_021822 | APOBEC3G | apolipoprotein B mRNA editing enzyme, catalytic polypeptide-like 3G | 1.8 |
| NM_012383 | OSTF1 | osteoclast stimulating factor 1 | 1.8 |
| OTTHUMT00000060170 | CHST12 | carbohydrate (chondroitin 4) sulfotransferase 12 | 1.8 |
| NM_001017373 | SAMD3 | sterile alpha motif domain containing 3 | 1.8 |
| NM_024312 | GNPTAB | N-acetylglucosamine-1-phosphate transferase, alpha and beta subunits | 1.8 |
| NM_001286152 | MPZL3 | myelin protein zero-like 3 | 1.8 |
| NM_017629 | AGO4 | argonaute RISC catalytic component 4 | 1.8 |
| NM_133171 | ELMO2 | engulfment and cell motility 2 | 1.8 |
| NM_001042685 | LGALS9B | lectin, galactoside-binding, soluble, 9B | 1.8 |
| NM_001145335 | UBE2Q2 | ubiquitin-conjugating enzyme E2Q family member 2 | 1.8 |
| NM_015230 | ARAP2 | ArfGAP with RhoGAP domain, ankyrin repeat and PH domain 2 | 1.8 |
| NM_000660 | TGFB1 | transforming growth factor beta 1 | 1.7 |
| NM_001290023 | IL12RB1 | interleukin 12 receptor, beta 1 | 1.7 |
| NM_001004470 | ST8SIA6 | ST8 alpha-N-acetyl-neuraminide alpha-2,8-sialyltransferase 6 | 1.7 |
| NM_000527 | LDLR | low density lipoprotein receptor | 1.7 |
| NM_001270780 | GZMH | granzyme H | 1.7 |
| NM_001301029 | USP28 | ubiquitin specific peptidase 28 | 1.7 |
| NM_001291726 | PGAP3 | post-GPI attachment to proteins 3 | 1.7 |
| NM_001195059 | PLEKHO2 | pleckstrin homology domain containing, family O member 2 | 1.7 |
| NM_005686 | SOX13 | SRY box 13 | 1.7 |
| NM_001282862 | RASGEF1A | RasGEF domain family member 1A | 1.7 |
| NM_006272 | S100B | S100 calcium binding protein B | 1.7 |
| NM_004155 | SERPINB9 | serpin peptidase inhibitor, clade B (ovalbumin), member 9 | 1.7 |
| NM_020820 | PREX1 | phosphatidylinositol-3,4,5-trisphosphate-dependent Rac exchange factor 1 | 1.7 |
| NR_046400 | DIP2A-IT1 | DIP2A intronic transcript 1 | 1.7 |
| XR_926994 | LOC105375130 | uncharacterized LOC105375130 | 1.7 |
| NR_029683 | MIR142 | microRNA 142 | 1.7 |
| NR_003003 | SCARNA17 | small Cajal body-specific RNA 17 | 1.7 |
| NM_001270507 | TNFAIP3 | tumor necrosis factor, alpha-induced protein 3 | 1.6 |
| NM_022365 | DNAJC1 | DnaJ (Hsp40) homolog, subfamily C, member 1 | 1.6 |
| NM_001278736 | CCL5 | chemokine (C-C motif) ligand 5 | 1.6 |
| NM_001199805 | KLRC4-KLRK1 | KLRC4-KLRK1 readthrough | 1.6 |
| NM_001286999 | ST6GALNAC6 | ST6 (alpha-N-acetyl-neuraminyl-2,3-beta-galactosyl-1,3)-N-acetylgalactosaminide alpha-2,6-sialyltransferase 6 | 1.6 |
| AK123224 | LOC100506928 | uncharacterized LOC100506928 | 1.6 |
| X58060 | SNORD13P2 | small nucleolar RNA, C/D box 13 pseudogene 2 | 1.6 |
| NM_001114380 | ITGAL | integrin alpha L | 1.5 |
