# Supplementary material for: Clinical significance of CD161+CD4+ T cells in the development of chronic antibody-mediated rejection in kidney transplant recipients
Source: PLoS One. 2018 Jul 16;13(7):e0200631. doi: 10.1371/journal.pone.0200631 (PMC6047803; doi:10.1371/journal.pone.0200631)
Supplement: S2 Table — (DOCX) [file pone.0200631.s003.docx]

**S2 Table. Down-regulated genes in CD161^+^ T cells compared with CD161^-^ T cells.**

| **Gene Accession** | **Gene_Symbol** | **Gene Description** | **FC** |
| --- | --- | --- | --- |
| AB087877 | ZCWPW2 | zinc finger, CW type with PWWP domain 2 | -32.1 |
| AK090461 | IGHD | immunoglobulin heavy constant delta | -27.0 |
| OTTHUMT00000325664 | IGHV1-18 | immunoglobulin heavy variable 1-18 | -26.6 |
| NM_144646 | JCHAIN | joining chain of multimeric IgA and IgM | -24.2 |
| OTTHUMT00000325171 | IGHV3-33 | immunoglobulin heavy variable 3-33 | -24.0 |
| OTTHUMT00000320966 | IGLC7 | immunoglobulin lambda constant 7 | -20.9 |
| OTTHUMT00000321818 | IGLC2 | immunoglobulin lambda constant 2 (Kern-Oz- marker) | -19.0 |
| NM_001185099 | CD22 | CD22 molecule | -16.8 |
| AF035023 | IGHV3-48 | immunoglobulin heavy variable 3-48 | -16.4 |
| AF067420 | IGHA1 | immunoglobulin heavy constant alpha 1 | -15.3 |
| OTTHUMT00000323404 | IGKV2-24 | immunoglobulin kappa variable 2-24 | -15.0 |
| AF035031 | IGKV3D-15 | immunoglobulin kappa variable 3D-15 (gene/pseudogene) | -14.3 |
| NM_021950 | MS4A1 | membrane-spanning 4-domains, subfamily A, member 1 | -13.3 |
| NM_001083907 | BANK1 | B-cell scaffold protein with ankyrin repeats 1 | -13.3 |
| AK057754 | IGHG1 | immunoglobulin heavy constant gamma 1 (G1m marker) | -11.7 |
| OTTHUMT00000325168 | IGHV4-34 | immunoglobulin heavy variable 4-34 | -11.5 |
| NM_001286761 | KIAA0226L | KIAA0226-like | -10.3 |
| NM_001159397 | FCRL1 | Fc receptor-like 1 | -9.5 |
| ENST00000610952 | CD24 | CD24 molecule | -9.1 |
| BC012159 | IGLC1 | immunoglobulin lambda constant 1 (Mcg marker) | -8.6 |
| BC066343 | IGKC | immunoglobulin kappa constant | -8.6 |
| NM_052847 | GNG7 | guanine nucleotide binding protein (G protein), gamma 7 | -8.4 |
| NM_001783 | CD79A | CD79a molecule, immunoglobulin-associated alpha | -8.3 |
| NM_005582 | CD180 | CD180 molecule | -7.7 |
| NR_024433 | LINC00926 | long intergenic non-protein coding RNA 926 | -7.6 |
| OTTHUMT00000325683 | IGHV3-13 | immunoglobulin heavy variable 3-13 | -7.6 |
| NR_039760 | MIR378I | microRNA 378i | -7.0 |
| NM_001280547 | PAX5 | paired box 5 | -6.7 |
| AK090464 | IGHM | immunoglobulin heavy constant mu | -6.4 |
| NM_020945 | WDFY4 | WDFY family member 4 | -6.1 |
| NM_019111 | HLA-DRA | major histocompatibility complex, class II, DR alpha | -6.0 |
| NM_001114094 | BLNK | B-cell linker | -5.9 |
| OTTHUMT00000323290 | IGKV2D-24 | immunoglobulin kappa variable 2D-24 (non-functional) | -5.9 |
| NM_001099658 | LRRN3 | leucine rich repeat neuronal 3 | -5.8 |
| NM_001286247 | RALGPS2 | Ral GEF with PH domain and SH3 binding motif 2 | -5.8 |
| OTTHUMT00000326390 | IGHG4 | immunoglobulin heavy constant gamma 4 (G4m marker) | -5.7 |
| XR_915947 | LOC105370652 | uncharacterized LOC105370652 | -5.6 |
| NM_001159488 | FCRL2 | Fc receptor-like 2 | -5.2 |
| NR_026800 | KIAA0125 | KIAA0125 | -4.9 |
| NM_001271842 | SCIMP | SLP adaptor and CSK interacting membrane protein | -4.9 |
| NM_018014 | BCL11A | B-cell CLL/lymphoma 11A (zinc finger protein) | -4.8 |
| NM_001290360 | EBF1 | early B-cell factor 1 | -4.8 |
| NM_000246 | CIITA | class II, major histocompatibility complex, transactivator | -4.8 |
| NM_013943 | CLIC4 | chloride intracellular channel 4 | -4.6 |
| NR_029373 | LEF1-AS1 | LEF1 antisense RNA 1 | -4.6 |
| NM_001184866 | FCRLA | Fc receptor-like A | -4.6 |
| OTTHUMT00000324606 | IGHV5-51 | immunoglobulin heavy variable 5-51 | -4.5 |
| AK123975 | IGH | immunoglobulin heavy locus | -4.5 |
| NM_001304428 | GAPT | GRB2-binding adaptor protein, transmembrane | -4.3 |
| BC065820 | IGHV4-31 | immunoglobulin heavy variable 4-31 | -4.3 |
| NM_001178098 | CD19 | CD19 molecule | -4.2 |
| NM_005450 | NOG | noggin | -4.2 |
| NR_003187 | NCF1C | neutrophil cytosolic factor 1C pseudogene | -4.1 |
| XR_246553 | LOC101926893 | uncharacterized LOC101926893 | -4.1 |
| NM_001250 | CD40 | CD40 molecule, TNF receptor superfamily member 5 | -3.9 |
| NM_000397 | CYBB | cytochrome b-245, beta polypeptide | -3.8 |
| NR_003186 | NCF1B | neutrophil cytosolic factor 1B pseudogene | -3.8 |
| OTTHUMT00000469136 | IGHV3OR16-7 | immunoglobulin heavy variable 3/OR16-7 (pseudogene) | -3.8 |
| NM_001130713 | LEF1 | lymphoid enhancer-binding factor 1 | -3.8 |
| NM_001304351 | ADAM28 | ADAM metallopeptidase domain 28 | -3.8 |
| NM_005160 | ADRBK2 | adrenergic, beta, receptor kinase 2 | -3.7 |
| NM_015130 | TBC1D9 | TBC1 domain family, member 9 (with GRAM domain) | -3.6 |
| NM_001017388 | TLR10 | toll-like receptor 10 | -3.5 |
| OTTHUMT00000321836 | IGLV2-18 | immunoglobulin lambda variable 2-18 | -3.5 |
| OTTHUMT00000323398 | IGKV1-16 | immunoglobulin kappa variable 1-16 | -3.5 |
| NM_005615 | RNASE6 | ribonuclease, RNase A family, k6 | -3.5 |
| NM_001037535 | SCML1 | sex comb on midleg-like 1 (Drosophila) | -3.5 |
| AF035025 | IGHV1-69 | immunoglobulin heavy variable 1-69 | -3.5 |
| NM_001098524 | FAM129C | family with sequence similarity 129, member C | -3.5 |
| NM_001168647 | CNKSR2 | connector enhancer of kinase suppressor of Ras 2 | -3.4 |
| NM_001256850 | TTN | titin | -3.4 |
| NM_001301714 | CCR7 | chemokine (C-C motif) receptor 7 | -3.4 |
| NM_004271 | LY86 | lymphocyte antigen 86 | -3.4 |
| NM_012108 | STAP1 | signal transducing adaptor family member 1 | -3.4 |
| OTTHUMT00000324210 | IGHV3-72 | immunoglobulin heavy variable 3-72 | -3.4 |
| OTTHUMT00000324613 | IGHV3-49 | immunoglobulin heavy variable 3-49 | -3.3 |
| NM_001297714 | SWAP70 | SWAP switching B-cell complex 70kDa subunit | -3.3 |
| NM_001139488 | RASGRP3 | RAS guanyl releasing protein 3 (calcium and DAG-regulated) | -3.3 |
| NM_001006658 | CR2 | complement component (3d/Epstein Barr virus) receptor 2 | -3.2 |
| NM_001029884 | PLEKHG1 | pleckstrin homology domain containing, family G (with RhoGef domain) member 1 | -3.2 |
| NR_002798 | NAPSB | napsin B aspartic peptidase, pseudogene | -3.2 |
| NM_001102 | ACTN1 | actinin, alpha 1 | -3.2 |
| NM_001145107 | NELL2 | neural EGFL like 2 | -3.2 |
| NM_052945 | TNFRSF13C | tumor necrosis factor receptor superfamily, member 13C | -3.1 |
| OTTHUMT00000321830 | IGLV3-19 | immunoglobulin lambda variable 3-19 | -3.1 |
| NM_003679 | KMO | kynurenine 3-monooxygenase (kynurenine 3-hydroxylase) | -3.1 |
| NM_002118 | HLA-DMB | major histocompatibility complex, class II, DM beta | -3.1 |
| OTTHUMT00000326208 | IGHD2-15 | immunoglobulin heavy diversity 2-15 | -3.1 |
| NM_001195388 | FCRL5 | Fc receptor-like 5 | -3.1 |
| NM_001025108 | AFF3 | AF4/FMR2 family, member 3 | -3.1 |
| OTTHUMT00000323357 | IGKV3-11 | immunoglobulin kappa variable 3-11 | -3.1 |
| AF113887 | IGKC | immunoglobulin kappa constant | -3.0 |
| NM_001190981 | IL6ST | interleukin 6 signal transducer | -3.0 |
| NM_001204118 | CLEC17A | C-type lectin domain family 17, member A | -3.0 |
| NM_001085357 | BTLA | B and T lymphocyte associated | -3.0 |
| NM_016562 | TLR7 | toll-like receptor 7 | -2.9 |
| NM_001293274 | CACHD1 | cache domain containing 1 | -2.9 |
| NM_001170794 | BACH2 | BTB and CNC homology 1, basic leucine zipper transcription factor 2 | -2.9 |
| NM_001039396 | MPEG1 | macrophage expressed 1 | -2.8 |
| ENST00000360669 | FAM153A | family with sequence similarity 153, member A | -2.8 |
| NM_000032 | ALAS2 | 5-aminolevulinate synthase 2 | -2.8 |
| NM_001144952 | SDK2 | sidekick cell adhesion molecule 2 | -2.8 |
| NM_000558 | HBA1 | hemoglobin, alpha 1 | -2.8 |
| NM_000698 | ALOX5 | arachidonate 5-lipoxygenase | -2.8 |
| OTTHUMT00000323135 | IGKV1-5 | immunoglobulin kappa variable 1-5 | -2.7 |
| NM_001080824 | TRABD2A | TraB domain containing 2A | -2.7 |
| NM_020404 | CD248 | CD248 molecule, endosialin | -2.7 |
| NR_119378 | LOC102723373 | uncharacterized LOC102723373 | -2.6 |
| OTTHUMT00000323476 | IGKV1-39 | immunoglobulin kappa variable 1-39 (gene/pseudogene) | -2.6 |
| NM_004948 | DSC1 | desmocollin 1 | -2.6 |
| NM_000626 | CD79B | CD79b molecule, immunoglobulin-associated beta | -2.6 |
| NM_001005328 | OR2A7 | olfactory receptor, family 2, subfamily A, member 7 | -2.6 |
| NM_001715 | BLK | BLK proto-oncogene, Src family tyrosine kinase | -2.6 |
| NM_002120 | HLA-DOB | major histocompatibility complex, class II, DO beta | -2.6 |
| NM_001308339 | DENND5B | DENN/MADD domain containing 5B | -2.6 |
| NM_014978 | SORCS3 | sortilin-related VPS10 domain containing receptor 3 | -2.6 |
| NM_001271606 | BASP1 | brain abundant, membrane attached signal protein 1 | -2.5 |
| NM_003498 | SNN | stannin | -2.5 |
| NM_005534 | IFNGR2 | interferon gamma receptor 2 (interferon gamma transducer 1) | -2.5 |
| NM_000061 | BTK | Bruton agammaglobulinemia tyrosine kinase | -2.5 |
| NM_002122 | HLA-DQA1 | major histocompatibility complex, class II, DQ alpha 1 | -2.5 |
| OTTHUMT00000352519 | TRBV30 | T cell receptor beta variable 30 (gene/pseudogene) | -2.5 |
| NM_001129778 | GRAPL | GRB2-related adaptor protein-like | -2.5 |
| OTTHUMT00000410946 | TRAJ52 | T cell receptor alpha joining 52 | -2.4 |
| NM_000517 | HBA2 | hemoglobin, alpha 2 | -2.4 |
| NM_173511 | FAM117B | family with sequence similarity 117, member B | -2.4 |
| NM_001195286 | IRF4 | interferon regulatory factor 4 | -2.4 |
| NM_002230 | JUP | junction plakoglobin | -2.4 |
| NM_001289010 | MAN1C1 | mannosidase, alpha, class 1C, member 1 | -2.4 |
| XR_938631 | LOC105377225 | uncharacterized LOC105377225 | -2.4 |
| NM_001025158 | CD74 | CD74 molecule, major histocompatibility complex, class II invariant chain | -2.4 |
| BC073773 | IGHV4-31 | immunoglobulin heavy variable 4-31 | -2.4 |
| NM_001256482 | EPHX2 | epoxide hydrolase 2, cytoplasmic | -2.4 |
| BC007304 | LOC84843 | uncharacterized LOC84843 | -2.4 |
| NM_022336 | EDAR | ectodysplasin A receptor | -2.4 |
| NM_018456 | EAF2 | ELL associated factor 2 | -2.4 |
| NM_006613 | GRAP | GRB2-related adaptor protein | -2.4 |
| NM_016591 | GCNT4 | glucosaminyl (N-acetyl) transferase 4, core 2 | -2.4 |
| NM_001265615 | FAM153B | family with sequence similarity 153, member B | -2.3 |
| OTTHUMT00000321841 | IGLV2-11 | immunoglobulin lambda variable 2-11 | -2.3 |
| NM_001127370 | CDCA7L | cell division cycle associated 7-like | -2.3 |
| OTTHUMT00000477584 | TRAJ36 | T cell receptor alpha joining 36 | -2.3 |
| NM_001131005 | MEF2C | myocyte enhancer factor 2C | -2.3 |
| NM_012093 | AK5 | adenylate kinase 5 | -2.3 |
| NM_001098725 | TCL1A | T-cell leukemia/lymphoma 1A | -2.3 |
| NM_001206924 | CD86 | CD86 molecule | -2.3 |
| NM_002371 | MAL | mal, T-cell differentiation protein | -2.3 |
| NM_001306151 | DAPP1 | dual adaptor of phosphotyrosine and 3-phosphoinositides | -2.3 |
| M87866 | OR6C4 | olfactory receptor, family 6, subfamily C, member 4 | -2.3 |
| NM_001257291 | SLC9A7 | solute carrier family 9, subfamily A (NHE7, cation proton antiporter 7), member 7 | -2.3 |
| NR_002915 | SNORA74A | small nucleolar RNA, H/ACA box 74A | -2.3 |
| ENST00000499006 | LINC01550 | long intergenic non-protein coding RNA 1550 | -2.3 |
| OTTHUMT00000409911 | TRAV26-2 | T cell receptor alpha variable 26-2 | -2.3 |
| NM_020805 | KLHL14 | kelch-like family member 14 | -2.3 |
| OTTHUMT00000410950 | TRAJ48 | T cell receptor alpha joining 48 | -2.3 |
| NM_001173977 | LRRC16A | leucine rich repeat containing 16A | -2.3 |
| NR_103776 | CHRM3-AS2 | CHRM3 antisense RNA 2 | -2.3 |
| NM_001174060 | OSBPL10 | oxysterol binding protein-like 10 | -2.2 |
| XR_947770 | LOC105378943 | uncharacterized LOC105378943 | -2.2 |
| NM_003840 | TNFRSF10D | tumor necrosis factor receptor superfamily, member 10d, decoy with truncated death domain | -2.2 |
| NM_173558 | FGD2 | FYVE, RhoGEF and PH domain containing 2 | -2.2 |
| OTTHUMT00000410953 | TRAJ45 | T cell receptor alpha joining 45 | -2.2 |
| NR_126375 | LINC01336 | long intergenic non-protein coding RNA 1336 | -2.2 |
| NM_000418 | IL4R | interleukin 4 receptor | -2.2 |
| OTTHUMT00000323399 | IGKV1-17 | immunoglobulin kappa variable 1-17 | -2.2 |
| OTTHUMT00000410959 | TRAJ39 | T cell receptor alpha joining 39 | -2.2 |
| OTTHUMT00000410960 | TRAJ38 | T cell receptor alpha joining 38 | -2.2 |
| NM_006120 | HLA-DMA | major histocompatibility complex, class II, DM alpha | -2.2 |
| NM_001207019 | FCER2 | Fc fragment of IgE, low affinity II, receptor for (CD23) | -2.2 |
| NM_001271594 | SESN3 | sestrin 3 | -2.2 |
| BC073782 | IGHG1 | immunoglobulin heavy constant gamma 1 (G1m marker) | -2.2 |
| NM_001316745 | CCDC141 | coiled-coil domain containing 141 | -2.1 |
| OTTHUMT00000323134 | IGKV1-6 | immunoglobulin kappa variable 1-6 | -2.1 |
| NM_003196 | TCEA3 | transcription elongation factor A (SII), 3 | -2.1 |
| XM_011539990 | WDFY4 | WDFY family member 4 | -2.1 |
| AY358248 | LOC100131541 | uncharacterized LOC100131541 | -2.1 |
| AK097859 | IGHM | immunoglobulin heavy constant mu | -2.1 |
| NM_001040107 | HVCN1 | hydrogen voltage gated channel 1 | -2.1 |
| XR_939453 | LOC105377538 | uncharacterized LOC105377538 | -2.1 |
| NM_001287005 | SUSD3 | sushi domain containing 3 | -2.1 |
| NR_002157 | OR2A9P | olfactory receptor, family 2, subfamily A, member 9 pseudogene | -2.1 |
| NM_152653 | UBE2E2 | ubiquitin conjugating enzyme E2E 2 | -2.1 |
| NM_017593 | BMP2K | BMP2 inducible kinase | -2.1 |
| XR_920027 | LOC105372589 | uncharacterized LOC105372589 | -2.1 |
| NR_039643 | MIR4441 | microRNA 4441 | -2.1 |
| NM_001032998 | KYNU | kynureninase | -2.1 |
| NM_001243254 | DENND5A | DENN/MADD domain containing 5A | -2.1 |
| NM_207468 | FAM177B | family with sequence similarity 177, member B | -2.0 |
| NM_001135109 | RIC3 | RIC3 acetylcholine receptor chaperone | -2.0 |
| NR_024075 | ADGRE4P | adhesion G protein-coupled receptor E4, pseudogene | -2.0 |
| NM_016582 | SLC15A3 | solute carrier family 15 (oligopeptide transporter), member 3 | -2.0 |
| OTTHUMT00000325673 | IGHV3-20 | immunoglobulin heavy variable 3-20 | -2.0 |
| NM_001560 | IL13RA1 | interleukin 13 receptor, alpha 1 | -2.0 |
| NR_002974 | SNORA80E | small nucleolar RNA, H/ACA box 80E | -2.0 |
| NM_001130020 | ATP6V0A1 | ATPase, H+ transporting, lysosomal V0 subunit a1 | -2.0 |
| NM_006868 | RAB31 | RAB31, member RAS oncogene family | -2.0 |
| NR_026677 | MIR600HG | MIR600 host gene | -2.0 |
| NM_001308174 | PLD4 | phospholipase D family, member 4 | -2.0 |
| NM_001256732 | SSBP2 | single-stranded DNA binding protein 2 | -2.0 |
| NM_024798 | SNX22 | sorting nexin 22 | -2.0 |
| NM_014399 | TSPAN13 | tetraspanin 13 | -2.0 |
| NM_000484 | APP | amyloid beta (A4) precursor protein | -1.9 |
| NM_152272 | CHMP7 | charged multivesicular body protein 7 | -1.9 |
| NM_001286455 | SLC22A23 | solute carrier family 22, member 23 | -1.9 |
| NM_001134398 | VAV2 | vav 2 guanine nucleotide exchange factor | -1.9 |
| NR_002911 | SNORA71A | small nucleolar RNA, H/ACA box 71A | -1.9 |
| NM_000565 | IL6R | interleukin 6 receptor | -1.9 |
| ENST00000318010 | GANC | glucosidase, alpha; neutral C | -1.9 |
| NR_033309 | SNORA70F | small nucleolar RNA, H/ACA box 70F | -1.9 |
| NM_002737 | PRKCA | protein kinase C, alpha | -1.9 |
| NM_004625 | WNT7A | wingless-type MMTV integration site family, member 7A | -1.9 |
| XR_926056 | LOC105374778 | uncharacterized LOC105374778 | -1.9 |
| NM_001010919 | FAM26F | family with sequence similarity 26, member F | -1.9 |
| XR_914813 | LOC105370125 | uncharacterized LOC105370125 | -1.9 |
| NM_001012505 | FOXP1 | forkhead box P1 | -1.9 |
| AK127936 | TRAV20 | T cell receptor alpha variable 20 | -1.9 |
| NM_032427 | MAML2 | mastermind-like transcriptional coactivator 2 | -1.9 |
| NM_024669 | ANKRD55 | ankyrin repeat domain 55 | -1.9 |
| NM_000655 | SELL | selectin L | -1.8 |
| NM_001251977 | RCAN3 | RCAN family member 3 | -1.8 |
| NM_001190259 | GCSAM | germinal center-associated, signaling and motility | -1.8 |
| NM_002163 | IRF8 | interferon regulatory factor 8 | -1.8 |
| NM_001278549 | PDK1 | pyruvate dehydrogenase kinase, isozyme 1 | -1.8 |
| NR_036521 | ZNF667-AS1 | ZNF667 antisense RNA 1 (head to head) | -1.8 |
| NM_001285549 | ZDBF2 | zinc finger, DBF-type containing 2 | -1.8 |
| BC020240 | IGHM | immunoglobulin heavy constant mu | -1.8 |
| NR_039602 | MIR378D2 | microRNA 378d-2 | -1.8 |
| NM_001270942 | KLF7 | Kruppel-like factor 7 (ubiquitous) | -1.8 |
| BC025727 | TRAV20 | T cell receptor alpha variable 20 | -1.8 |
| OTTHUMT00000410948 | TRAJ50 | T cell receptor alpha joining 50 | -1.8 |
| NM_000922 | PDE3B | phosphodiesterase 3B, cGMP-inhibited | -1.8 |
| BC039714 | TRDV2 | T cell receptor delta variable 2 | -1.8 |
| XR_930387 | LOC105376290 | uncharacterized LOC105376290 | -1.7 |
| OTTHUMT00000410964 | TRAJ34 | T cell receptor alpha joining 34 | -1.7 |
| XR_429044 | LOC101060038 | uncharacterized LOC101060038 | -1.7 |
| OTTHUMT00000410975 | TRAJ23 | T cell receptor alpha joining 23 | -1.7 |
| OTTHUMT00000477580 | TRAJ37 | T cell receptor alpha joining 37 | -1.7 |
| OTTHUMT00000351233 | TRBV9 | T cell receptor beta variable 9 | -1.7 |
| NM_001170553 | VSIG1 | V-set and immunoglobulin domain containing 1 | -1.7 |
| OTTHUMT00000410942 | TRAJ56 | T cell receptor alpha joining 56 | -1.7 |
| NM_016224 | SNX9 | sorting nexin 9 | -1.7 |
| OTTHUMT00000410955 | TRAJ43 | T cell receptor alpha joining 43 | -1.6 |
| OTTHUMT00000410963 | TRAJ35 | T cell receptor alpha joining 35 (non-functional) | -1.6 |
| NR_030347 | MIR548C | microRNA 548c | -1.6 |
